# Healthy lifestyles, systemic inflammation and breast cancer risk: a mediation analysis

**DOI:** 10.1186/s12885-024-11931-5

**Published:** 2024-02-15

**Authors:** Yanyu Zhang, Mengjie Song, Zixuan Yang, Xiaoxi Huang, Yuxiang Lin, Haomin Yang

**Affiliations:** 1 Department of Epidemiology and Health Statistics, School of Public Health, Fujian Medical University, Xue Yuan Road 1, University Town, 350122 Fuzhou, China; 2 Department of Breast, Fujian Maternity and Child Health Hospital, College of Clinical Medicine for Obstetrics and Gynecology and Pediatrics, Fujian Medical University, Fuzhou, 350001 China; 3 Department of Breast Surgery, Fujian Medical University Union Hospital, Fuzhou, 350001 China; 4 Department of General Surgery, Fujian Medical University Union Hospital, Fuzhou, 350001 China; 5 Breast Cancer Institute, Fujian Medical University, Fuzhou, 350001 China; 6 Department of Medical Epidemiology and Biostatistics, Karolinska Institutet, Stockholm, 17177 Sweden

**Keywords:** HLI, Inflammation markers, Breast cancer, Mediators

## Abstract

**Background:**

Despite the known association between healthy lifestyles and reduced risk of breast cancer, it remains unclear whether systemic inflammation, as a consequence of unhealthy lifestyles, may mediate the association.

**Methods:**

A cohort study of 259,435 female participants in the UK Biobank was conducted to estimate hazard ratio (HR) for breast cancer according to 9 inflammation markers using Cox regression models. We further estimated the percentage of total association between healthy lifestyle index (HLI) and breast cancer that is mediated by these inflammation markers.

**Results:**

During 2,738,705 person-years of follow-up, 8,889 cases of breast cancer were diagnosed among 259,435 women in the UK Biobank cohort. Higher level of C-reactive protein (CRP), systemic immune-inflammation index (SII), CRP-to-albumin Ratio (CAR), CRP-to-lymphocyte Ratio (CLR), monocyte-to-HDL-c ratio (MHR), and neutrophil-to-HDL-c ratio (NHR) were associated with increased breast cancer risk, while a higher lymphocyte-to-monocyte ratio (LMR) was associated with a lower risk. The inverse association between HLI and breast cancer was weakly mediated by CRP (8.5%), SII (1.71%), CAR (8.66%), CLR (6.91%), MHR (6.27%), and NHR (7.33%). When considering individual lifestyle factors, CRP and CAR each mediated 16.58% and 17.20%, respectively, of the associations between diet score and breast cancer risk, while the proportion mediated for physical activity and breast cancer were 12.13% and 11.48%, respectively. Furthermore, MHR was found to mediate 13.84% and 12.01% of the associations between BMI, waist circumference, and breast cancer.

**Conclusion:**

The association of HLI and breast cancer is weakly mediated by the level of inflammation, particularly by CRP and CAR. Systemic inflammatory status may be an intermediate in the biological pathway of breast cancer development.

**Supplementary Information:**

The online version contains supplementary material available at 10.1186/s12885-024-11931-5.

## Introduction

Breast cancer is the most common cancer globally and the leading cause of cancer-related deaths among women. Known breast cancer risk factors include age, family history, menopausal status, genetics, as well as modifiable exposures such as reproductive and lifestyle-related factors (such as obesity, use of oral contraceptives, smoking, dietary patterns, and alcohol consumption) [1]. Unlike familial and genetic factors, which contribute less than 30% to the risk [2], breast cancer is more strongly associated with modifiable lifestyle factors [3]. Breast cancer could be avoided by removing unhealthy lifestyle factors, with the population attributable fraction being nearly 20% for BMI [4], about 10% for smoking [5], 8.98% for lack of whole grain intake [6], and 3.8% for insufficient physical activity [7]. Despite the known association between healthy lifestyles and breast cancer [8–11], the underlying mechanisms are still unclear.

Recent studies have indicated that inflammation plays a pivotal role in the development of several chronic diseases [12–14], including cancer [15]. Inflammation markers have been shown to be associated with cancer risk, including systemic immune-inflammation index (SII), neutrophil-to-lymphocyte ratio (NLR), platelet-to-lymphocyte ratio (PLR), and lymphocyte-to-monocyte ratio (LMR) [16]. However, there is limited evidence documenting their associations with the risk of breast cancer [16, 17]. Although C-reactive protein (CRP) was associated with breast cancer in many studies, the potential impact of several other inflammation markers on breast cancer risk remains unexplored, including neutrophils-to-HDL-c ratio (NHR), monocytes-to-HDL-c ratio (MHR), CRP-to-albumin ratio (CAR), and CRP-to-lymphocytes ratio (CLR).

The previous study showed that increasing physical activity and maintaining a healthy weight can effectively reduce inflammation in the body [18]. Apart from lifestyle factors, the classic Mediterranean dietary pattern, rich in vegetables, fruits, fish, nuts, etc., using olive oil as the main cooking oil can reduce cellular oxidative stress and inflammatory responses [19], while both smoking and excessive alcohol consumption can cause oxidative stress and inflammation [20, 21]. Given that inflammation has been linked to both lifestyle factors and the risk of breast cancer [22, 23], we propose that inflammation markers could serve as an intermediate marker of breast cancer risk. However, to date, no studies have examined the extent to which lifestyle factors influence the risk of breast cancer through their impact on inflammation. A better understanding of the role of inflammation markers may provide insights into the etiology of breast cancer, and may support interventions aimed at modifiable factors to reduce the incidence of breast cancer.

Therefore, to quantify the extent to which the associations between established lifestyle factors and breast cancer risk are mediated by inflammation markers, we analyzed the separate and joint effects of healthy lifestyle index (HLI) and these inflammation markers on breast cancer within the UK Biobank cohort. We further estimated the mediated effect for each component of HLI.

## Methods

### Study population

The UK Biobank (UKB) is a large prospective observational study, which recruited more than half a million participants (55% women) aged 40–69 years between 2006 and 2010 from 22 assessment centers. Comprehensive information was collected through self-administered touchscreen questionnaires and nurse-led interviews in the assessment centers, including sociodemographic data, lifestyle habits, dietary habits, medical history, and occupational and environmental exposure. The biological samples, including blood and urine, were collected from all participants during a physical examination. The participants were followed up to collect health-related information. Written informed consent was obtained from all participants. UKB has been reviewed by the North West Multi-center Research Ethics Committee and has implemented rigorous data quality control measures.

### Measurements of blood inflammation markers and their ratios

The blood samples collected were analyzed at the UK Biobank Centre laboratory within 24 h of the blood collection. Samples collected in a 4 ml vacuum containing EDTA (ethylene diamine tetraacetic acid) were analyzed using four Beckman Coulter LH750 instruments. Lymphocytes, neutrophils, and monocytes were quantified using a Differential/Complete Blood Count. Platelet count (PLT) was measured using the Coulter method. CRP and HDL-c measurements were conducted on a Beckman Coulter AU5800 using an Immuno-turbidimetric assay.

We selected blood inflammation markers, including NLR, SII, PLR, LMR, MHR, NHR, CRP, CAR, and CLR, based on previous studies that have suggested their associations with other cancers [16, 24]. The calculation methods for these markers are as follows: NLR = neutrophils/lymphocytes, SII = platelet count*(neutrophil count /lymphocyte count), PLR = platelet count / lymphocyte count, LMR = lymphocyte count / Monocyte count, MHR = Monocyte count / HDL-c, NHR = neutrophil count /HDL-c, CAR = CRP / albumin, CLR = CRP /lymphocyte count. CRP and HDL-c were detected directly in the blood. We, therefore, included women with completed information on these markers for further analysis.

### Ascertainment of healthy lifestyle Index

In this study, HLI was developed according to previous literature [25], incorporating factors such as diet, alcohol consumption, physical activity, body fat, and smoking. In UKB, a food frequency questionnaire was utilized to ask participants about the frequency and quantity of food intake over the previous 12 months. The validity and repeatability of the thirty-two-item food frequency questionnaire have been assessed and confirmed in a previous study [26]. Briefly, the questionnaire was validated using a web-based 24-hour dietary assessment, and its repeatability was assessed by having the same participants fill out the questionnaire again after a 4-year interval. The major food groups that have been studied for their potential carcinogenic properties included fruits, vegetables, grains, red meat (such as pork, beef, and lamb), and processed meat. Each item is scored on a scale of 0 to 0.5 points, and the total score ranges from 0 to 2. The frequency of alcohol consumption, physical activity, measurement of body fat (using body mass index (BMI) and Waist Circumference (WC)), and smoking are each assigned a score of 0 to 0.5 or 0 to 1, with the highest value (0.5 or 1) representing the highest category. The level of physical activity was evaluated by documenting the frequency and duration of walking, moderate-intensity, and vigorous-intensity exercises performed over the past week, using the International Physical Activity Questionnaire (IPAQ). According to the IPAQ scoring protocol (https://sites.google.com/site/theipaq/scoring-protocol), metabolic equivalent of task (MET) values of 8.0, 4.0, and 3.3 were assigned to vigorous physical activity, moderate physical activity, and walking, respectively [27]. The amount of physical activity per week (MET-minutes/week) was calculated by multiplying the duration and frequency of physical activities by the corresponding MET value [27]. The HLI was then constructed by summing up the scores for diet, alcohol consumption, physical activity, body fat (with a reversed core for premenopausal women, as obesity might be negatively associated with breast cancer risk in this group of women [28, 29]), and smoking. The construction of HLI is shown in Supplementary Table 1.

### Outcome ascertainment

The diagnosis of breast cancer was obtained through linkage to the National Health Service (NHS) Digital for England and Wales, and National Records of Scotland, NHS Central Register for Scotland by personal identification numbers, using the ICD-9 code 174 and ICD-10 code C50. Women who were diagnosed with breast cancer before participating in the UK biobank were excluded from the analysis, leaving 266,473 women in the study. Information regarding the cause and date of death was obtained via linkage to death registry records from National Health Service (NHS). Follow-up for the participants started from the date of enrollment and continued until the diagnosis of breast cancer, death, loss to follow-up, or the end of the study (December 31, 2019, taking into account the potential influence of the COVID-19 pandemic), whichever occurred first.

### Statistical analyses

We first used Cox proportional hazards models to estimate hazard ratios (HRs) for breast cancer according to the levels of inflammation markers by quartiles and as standardized continuous variables, with attained age as the underlying timescale. A trend test was utilized to examine the linear trend for the associations between inflammation markers and breast cancer risk. The basic model was adjusted for the UK Biobank Assessment Center, and the fully adjusted model was further adjusted for BMI, smoking, family history of breast cancer, oral contraceptive use, hormone replacement therapy, number of births, and age at menarche, and menopausal status at baseline. To account for false-positive findings caused by multiple testing, biomarkers with a *P-*value for trend < 0.05/9 (the Bonferroni-corrected threshold for 9 markers) were considered to be statistically significant. These associations were further tested through a stratified analysis by menopausal status, and within or beyond 2 years after the start of follow-up. Restricted cubic spline models were used to evaluate potential nonlinear relationships between inflammation markers and breast cancer.

In the first step of mediation analysis, we used linear regression to estimate the variations in blood inflammation markers associated with HLI and its components, while controlling for age at recruitment. Further mediation analyses were performed using the med4way package [30] for those inflammation markers significantly associated with HLI. The overall excess risk can be divided into four parts, including the controlled direct effect of HLI (explained only by HLI, and not by the inflammation markers), pure interaction (explained only by the interaction of HLI and inflammation markers), mediated interaction (explained by both the interaction and mediation effects of the inflammation markers), and pure indirect effects (explained only by the inflammation markers). The formula for calculating the percentage of mediation is as follows: (β_mediated interaction_ + β_indirect effect_)/(β_direct effect_ + β_interaction_ + β_mediated interaction_ + β_indirect effect_). In the mediation model, linear regression was used for the association between HLI and mediators, while Cox regression was used for the association between HLI and breast cancer. The analysis was adjusted for the UK Biobank Assessment Center, family history of breast cancer, number of births, oral contraceptive use, hormone replacement therapy, and age at menarche. In addition, sensitivity analyses stratified by menopausal status were also implemented. Body fatness (BMI and waist circumference) was excluded when constructing HLI in premenopausal women, while HLI with body fatness was used when we analyzed postmenopausal women. The same mediation analyses were also performed separately for each component of HLI. All statistical analyses were performed using Stata 17.

## Results

During 2,738,705 person-years of follow-up, 8,889 cases of breast cancer were diagnosed among 259,435 women in the UK Biobank cohort, corresponding to an incidence rate of 3.25 /1000 person-years. The characteristics of the study participants at baseline were presented in Table 1.

**Table 1:** Baseline characteristics of study participants in the UK Biobank

	Overall (*N* = 259,435)
Age at recruitment (years)	57 (8.01)
Menopausal status at recruitment	
Premenopausal	77,777 (29.98%)
Postmenopausal	181,658 (70.02%)
Body mass index (kg/m^2^)	
< 18.5	1957 (0.75%)
18.5–25.0	101,021 (38.94%)
25.0–30.0	94,871 (36.57%)
≥ 30.0	60,662 (23.38%)
Number of births	
0	48,304 (18.62%)
1	34,532 (13.31%)
2	113,359 (43.69%)
≥ 3	62,625 (24.14%)
Family history of breast cancer	
No	213,775 (82.4%)
Yes	26,774 (10.32%)
Age at menarche (years)	
< 13	97,778 (37.69%)
13–15	138,665 (53.45%)
≥ 15	14,778 (5.7%)
Oral contraceptive use	
No	48,521 (18.7%)
Yes	209,742 (80.85%)
Hormone replacement therapy	
No	159,779 (61.59%)
Yes	98,337 (37.9%)
Smoking	
Never	154,097 (59.4%)
Former	80,999 (31.22%)
Current	23,057 (8.89%)

### The association between inflammation markers and breast cancer

**Fig. 1:**
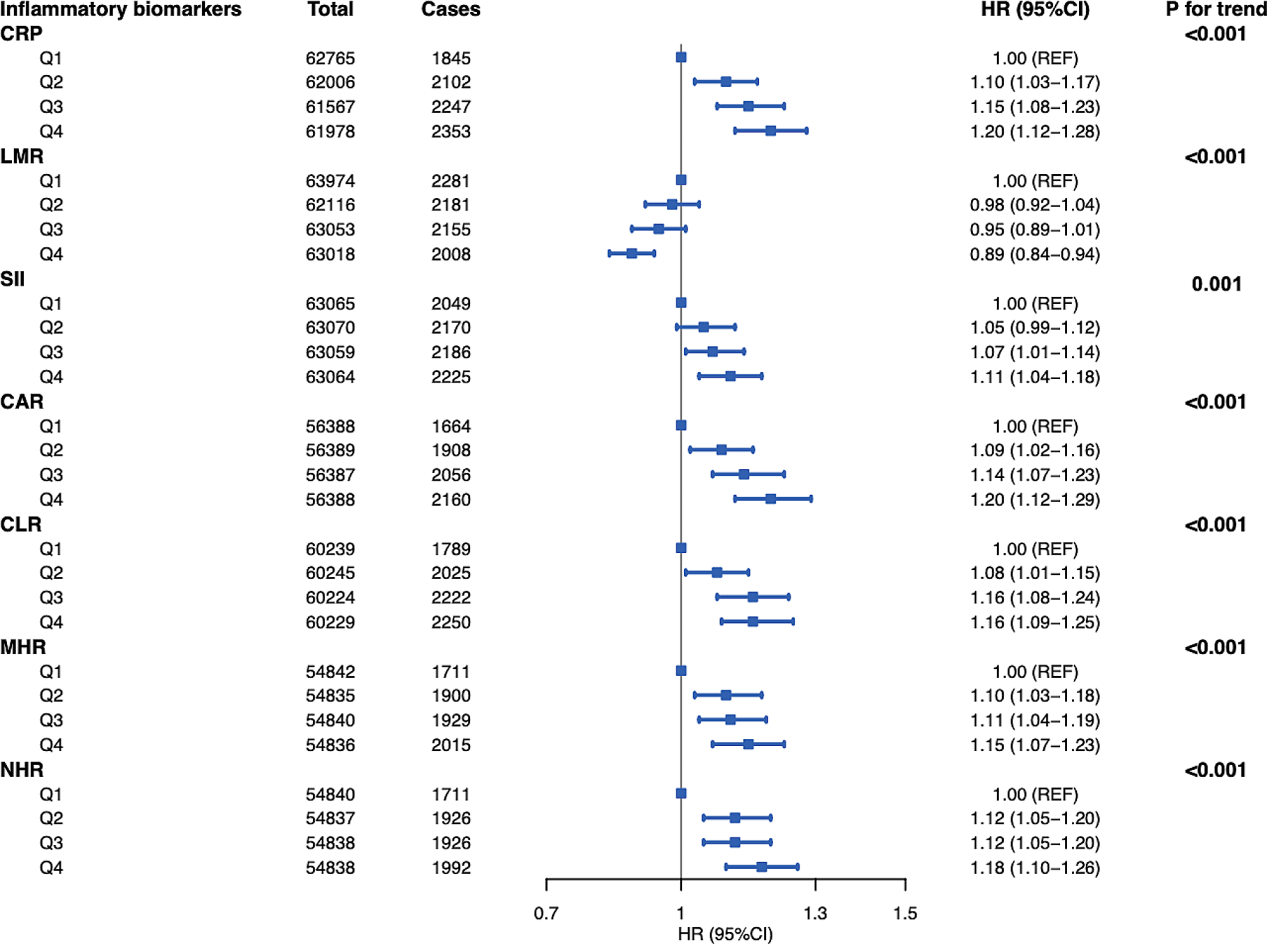
The forest plot for inflammation markers associated with the breast cancer risk. The multivariable Cox regression was performed to identify blood inflammation markers measured at baseline that may associate with the risk of breast cancer incidence. The fully adjusted model adjusted for UK Biobank assessment centers, BMI, smoking, family history of breast cancer, number of births, oral contraceptive use, hormone replacement therapy, age at menarche and menopausal status. Considering false-positive findings caused by multiple testing, biomarkers with *P* for trend < 0.05/9 (the Bonferroni corrected threshold) were considered statistically significant. The biomarkers significantly associated with breast cancer risk were shown in the forest plot, and the detailed results were provided in Supplementary Table 2

In the multivariable model, CRP, SII, CAR, CLR, MHR, and NHR were associated with an increased risk of breast cancer (for the highest quartile vs. lowest quartile, *HR*_CRP_=1.20, 95% *CI* = 1.12–1.28; *HR*_SII_ =1.11, 95% *CI* = 1.04–1.18; *HR*_CAR_ =1.20, 95% *CI* = 1.12–1.29; *HR*_CLR_ =1.16, 95% *CI* = 1.09–1.25, *HR*_MHR_ =1.15, 95% *CI* = 1.07–1.23; *HR*_NHR_ =1.18, 95% *CI* = 1.10–1.26), while LMR was inversely associated with breast cancer (*HR* = 0.89, 95% *CI* = 0.84–0.94), with all *P*-trend < 0.001(Fig. 1, Supplementary Table 2). An inverse U-shaped relationships between CRP, CAR and CLR, and breast cancer were also observed using cubic spline models (with p values for non-linearity of 0.0002, 0.0003, and 0.0035, respectively; Supplementary Fig. 1). NLR showed a significant positive association with breast cancer only in model 1, which was adjusted for assessment centers alone, while the association was not statistically significant in the multivariable adjusted model (Supplementary Table 2). In addition, we did not find any association between PLR and breast cancer risk.

In the sensitivity analysis stratified by menopausal status, the results remained unchanged for postmenopausal women, while we only observed statistically significant associations with LMR and MHR in premenopausal women (Supplementary Table 3). The results remain consistent even when starting the follow-up 2 years after recruitment. Detailed findings are available in Supplementary Table 4.

### The association between HLI and inflammation markers

With an increase of one standard deviation in HLI, there were varying degrees of reduction in CRP, SII, CAR, CLR, MHR, and NHR among these women, after adjusting for age at recruitment (*β*_CRP_*=*-0.150, 95%*CI=*-0.155, -0.145; *β*_SII_*=*-0.018, 95%*CI=*-0.020, -0.016; *β*_CAR_*=*-0.139, 95%*CI=*-0.143, -0.134; *β*_CLR_*=*-0.122, 95%*CI=*-0.127, -0.117; *β*_MHR_*=*-0.043, 95%*CI=*-0.046, -0.041; *β*_NHR_*=*-0.055, 95%*CI=*-0.057, -0.053, respectively, as shown in Supplementary Table 5), and these significant biomarkers were selected for further mediation analysis. The effects of each component of HLI, including diet score, physical activity, BMI, WC, and smoking were concordant, while the association with alcohol consumption was not statistically significant (Supplementary Table 6).

### The association between HLI and breast cancer

An increase of one standard deviation in HLI was associated with a 10% reduced risk of breast cancer (*HR* = 0.90, 95%*CI* = 0.88–0.93) (Supplementary Table 7). After adjusting for the inflammation markers as mediators, the effect of HLI changed slightly. The results did not differ appreciably when stratified by menopausal status (Supplementary Table 8). For each component of HLI, the risk of breast cancer was significantly increased among women with an alcohol intake of ≥ 5 times/week, a BMI ≥ 30 kg/m^2^, a waist circumference of ≥ 88 cm, and smoking (*HR*_*alcohol*_=1.15, 95%*CI* = 1.06–1.26; *HR*_*BMI*_=1.18, 95%*CI* = 1.12–1.25; *HR*_*WC*_ =1.22, 95%*CI* = 1.17–1.29; and *HR*_*smoking*_ =1.13, 95%*CI* = 1.05–1.22, respectively, Supplementary Table 9). Whereas, compared to women with physical activity levels of less than 600 MET-minutes/week, those with levels of at least 3000 MET-minutes/week had a 13% lower risk of breast cancer (*HR* = 0.87, 95%*CI* = 0.81–0.93).

### Mediating effects of inflammation markers on the association between HLI and breast cancer

**Fig. 2:**
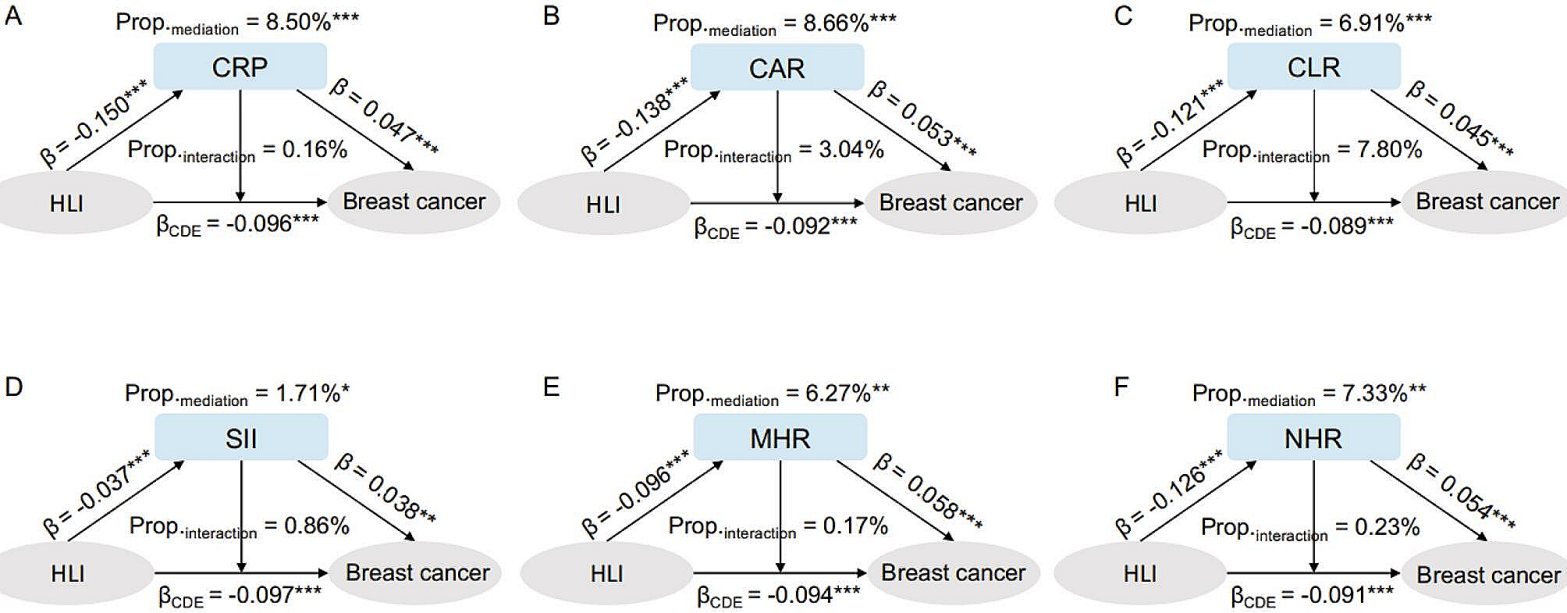
The associations between HLI, inflammation markers and breast cancer. The panel presents the relationship between HLI and breast cancer, and mediation through CRP, CAR, CLR, SII, MHR, NHR. Mediation effects are decomposed into controlled direct effects, pure interaction, mediated interaction, and pure indirect effects. The panel (**A**), (**B**), (**C**), (**D**), (**E**) and (**F**) present the results of mediation analyses between HLI, inflammation markers and breast cancer. Standardized coefficients, proportion of mediation and interaction are presented in the figure. ***indicates a *P* value < 0.001, ** indicates a *P* value < 0.01, * indicates a *P* value < 0.05. The detailed results are provided in Supplementary Table 10

In the mediation analysis, the pure indirect effects of HLI mediated by inflammation markers were as follows: -0.007 (95% CI: -0.010, -0.0035) for CRP, *− 0*.001 (95% CI: -0.002, -0.000) for SII, -0.007 (95% CI: -0.011, -0.004) for CAR, -0.005 (95% CI: -0.008, -0.003) for CLR, -0.006 (95% CI: -0.008, -0.003) for MHR, and − 0.007 (95% CI: -0.010, -0.034) for NHR. However, they only mediated 8.50%, 1.71%, 8.66%, 6.91%, 6.27%, and 7.33% of the inverse association between HLI and breast cancer risk (Fig. 2, Supplementary Table 10).

**Fig. 3:**
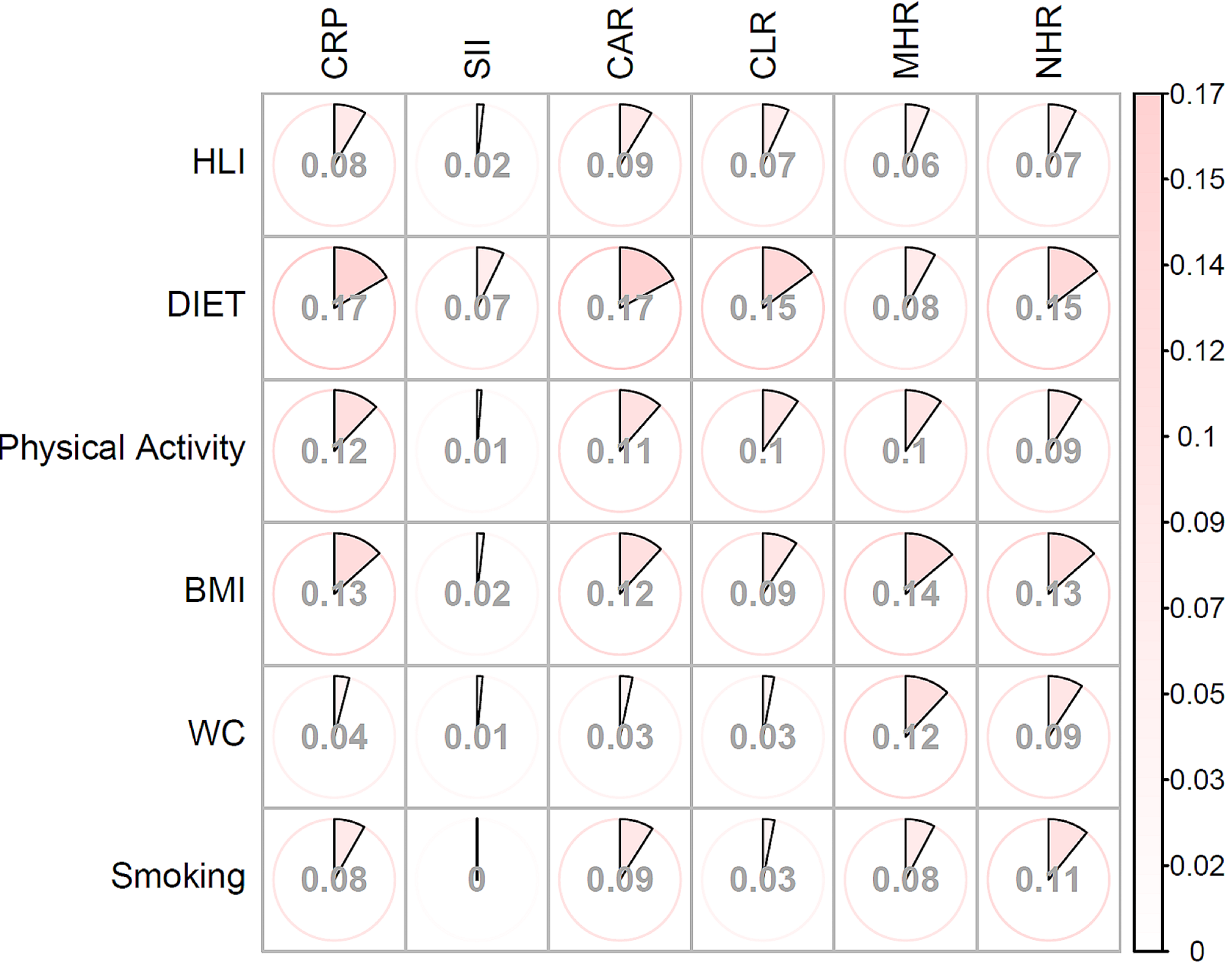
The percentage mediated by inflammation markers for the association between HLI and breast cancer, by components of HLI. The proportion of mediation is presented in the figure. The detailed results are provided in Supplementary Tables 11–15

For each component of HLI, the inverse associations between diet score and physical activity with breast cancer were largely mediated by CAR and CRP, with 16.58% and 17.20% of the overall excess risk for diet score, and 12.13% and 11.48% for physical activity, respectively (Fig. 3, Supplementary Tables 11–15). The association between lifestyle factors and breast cancer was not mediated by SII. In addition, MHR was found to mediate approximately 10% of the associations between each component of HLI and breast cancer. Specifically, it mediated 13.84% and 12.01% of the associations between BMI, waist circumference, and breast cancer.

## Discussion

In this large prospective cohort study, higher levels of CRP, SII, CAR, CLR, MHR, and NHR were associated with an increased risk of breast cancer, albeit LMR was inversely associated with breast cancer. These results persisted among postmenopausal women and among women who were followed 2 years after attendance. Mediation analyses indicated that the association between HLI and breast cancer was partially explained by the effects of these inflammation markers. Upon further investigation into the associations between components of HLI and breast cancer, we found that CRP and CAR mediated the associations between diet score and physical activity with breast cancer, while MHR mediated the effect of all the components of HLI on breast cancer.

In our study, novel inflammation markers related to CRP and HDL-c [24] were associated with breast cancer risk, while traditionally systemic inflammation markers such as PLR and NLR were not. In a recent study conducted in the UK Biobank, the associations between PLR, NLR, and breast cancer risk were not statistically significant either [16], which is consistent with our findings. Additionally, we also observed the dose-response effect of NHR and MHR using a cubic spline. Many of the biochemical processes altered during chronic inflammation are associated with tumorigenesis. Chronic inflammation can disrupt the homeostatic control of cellular signaling pathways, leading to precancerous changes or cell deterioration, and may also promote the incidence and development of breast cancer through mechanisms such as DNA damage, cell proliferation, and deregulation of epigenetic control [31]. Our findings on NHR, MHR, and breast cancer further emphasize the importance of metabolic inflammation in the development of breast cancer.

The association between CRP and breast cancer has been reported in previous studies, and further confirmed by a recent Mendelian randomization analysis [32]. However, we also found an inverted U-shaped relationship between CRP and breast cancer. The inverse association between CRP and breast cancer in the extremely high group could possibly be explained by the inverse association between breast cancer and autoimmune disease [33, 34], as these patients were characterized by high levels of CRP.

Several prior studies have indicated HLI and inflammation as independent factors for breast cancer [35]. In this study, we further estimated the extent to which the association between HLI and breast cancer is mediated by inflammation markers, with the largest extent of mediation through CAR. Among these markers, CRP and CAR also mediated the largest extent of the associations between diet score and physical activity with breast cancer. Previous studies have shown that physical activity [36] and consumption of fruit and vegetables [37] were inversely associated with CRP, CAR [38], and CLR, which is consistent with our results. These findings suggest that the diet and physical activity intervention at CRP, which targets chronic low-grade inflammation, may provide benefits for high-risk women [39].

In our study, MHR mediated the associations between all components of HLI and breast cancer. MHR has been identified as a reliable predictive biomarker for metabolic syndrome [40], while metabolic syndrome is often a result of an unhealthy lifestyle and is associated with an increased risk of breast cancer [41]. Interestingly, the mediating effects of MHR for BMI and WC were similar, indicating that there is no different metabolic process in central or gluteal adiposity in the onset of breast cancer through the pathway of metabolic inflammation. Taken together, our findings suggest that adopting a comprehensive healthy lifestyle including regular physical activity, diet, and no-smoking, could improve inflammation levels in the body, and reduce the risk of breast cancer.

### Strengths and limitations

This study has several advantages, including the comprehensive information collected in the UK biobank cohort, which is readily available for users to use, and the use of med4way to decompose the overall effect. This approach allowed for an examination of the extent to which lifestyle factors influence breast cancer risk through their effects on inflammation markers. Moreover, these biomarkers are convenient, affordable, and promising to use.

However, this study also has limitations. Some biomarkers, such as CRP, may reflect both acute and chronic inflammation, while the acute inflammation status can change over time, which can lead to potential misclassification. We assumed that this misclassification occurred randomly, and this bias might have attenuated the association estimates. In addition, many of the significant associations were found only in postmenopausal women in the stratified analysis, due to the restricted number of premenopausal women in the UKB cohort. Further studies are needed to find the role of inflammation markers in premenopausal women. It should be mentioned that the associations between HLI and breast cancer may vary depending on the subtypes of breast cancer, and the proportion mediated by inflammation markers may also differ accordingly. However, information on breast cancer subtypes is currently unavailable in the UKB dataset.

## Conclusion

In conclusion, our study has shown that a higher HLI is associated with a reduced risk of breast cancer. The association was weakly mediated by the level of inflammation, particularly by CRP and CAR. The systemic inflammatory status may be an intermediate in biological pathway for the development of breast cancer, which is related to unhealthy lifestyles.

### Electronic supplementary material

Below is the link to the electronic supplementary material.

**Supplementary Material 1: Supplementary Table 1.** The construction of HLI **Supplementary Table 2.** The levels of inflammation markers and the risk of breast cancer among women from the UK Biobank **Supplementary Table 3.** The levels of inflammation markers and the risk of breast cancer by menopausal status **Supplementary Table 4.** The levels of inflammation markers and the risk of breast cancer grouped by 2 years entering the cohort **Supplementary Table 5.** The associations between HLI and inflammation markers **Supplementary Table 6.** The associations between individual components of HLI and inflammation markers **Supplementary Table 7.** Independent and joint effects of HLI and inflammation markers on breast cancer risk **Supplementary Table 8.** The association between HLI and the risk of breast cancer by menopausal status **Supplementary Table 9.** The associations between individual components of HLI and breast cancer risk among overall, premenopausal, and postmenopausal women in UK Biobank **Supplementary Table 10.** The mediation analysis of the inflammation markers in the association between HLI and breast cancer risk **Supplementary Table 11.** Mediating effects of inflammation markers on the association between diet score and breast cancer risk **Supplementary Table 12.** Mediating effects of inflammation markers on the association between physical activity and breast cancer risk **Supplementary Table 13.** Mediating effects of inflammation markers on the association between BMI and breast cancer risk **Supplementary Table 14.** Mediating effects of inflammation markers on the association between WC and breast cancer risk **Supplementary Table 15.** Mediating effects of inflammation markers on the association between smoking and breast cancer risk **Supplementary Figure 1.** The associations between levels of CRP, LMR, SII, CAR, CLR, MHR and NHR and breast cancer were evaluated on a continuous scale with restricted cubic spline curves based on cox regression with four knots. Solid lines are multivariable adjusted odds ratios, with dashed lines showing 95% confidence intervals. Blue curves show the fraction of breast cancer with different levels of inflammation markers

## Data Availability

Data from the UK Biobank (http://www.ukbiobank.ac.uk/) are available to all researchers upon making an application. Part of this research was conducted using the UK Biobank Resource under Application 61083.
