# Supplementary material for: Healthy lifestyles, systemic inflammation and breast cancer risk: a mediation analysis
Source: BMC Cancer. 2024 Feb 15;24:208. doi: 10.1186/s12885-024-11931-5 (PMC10868083; doi:10.1186/s12885-024-11931-5)
Supplement: Supplementary file 1 — Supplementary Material 1: Supplementary Table 1. The construction of HLI Supplementary Table 2. The levels of inflammation markers and the risk of breast cancer among women from the UK Biobank Supplementary Table 3. The levels of inflammation markers and the risk of breast cancer by menopausal status Supplementary Table 4. The levels of inflammation markers and the risk of breast cancer grouped by 2 years entering the cohort Supplementary Table 5. The associations between HLI and inflammation markers Supplementary Table 6. The associations between individual components of HLI and inflammation markers Supplementary Table 7. Independent and joint effects of HLI and inflammation markers on breast cancer risk Supplementary Table 8. The association between HLI and the risk of breast cancer by menopausal status Supplementary Table 9. The associations between individual components of HLI and breast cancer risk among overall, premenopausal, and postmenopausal women in UK Biobank Supplementary Table 10. The mediation analysis of the inflammation markers in the association between HLI and breast cancer risk Supplementary Table 11. Mediating effects of inflammation markers on the association between diet score and breast cancer risk Supplementary Table 12. Mediating effects of inflammation markers on the association between physical activity and breast cancer risk Supplementary Table 13. Mediating effects of inflammation markers on the association between BMI and breast cancer risk Supplementary Table 14. Mediating effects of inflammation markers on the association between WC and breast cancer risk Supplementary Table 15. Mediating effects of inflammation markers on the association between smoking and breast cancer risk Supplementary Figure 1. The associations between levels of CRP, LMR, SII, CAR, CLR, MHR and NHR and breast cancer were evaluated on a continuous scale with restricted cubic spline curves based on cox regression with four knots. Solid lines are multivariable adj [file 12885_2024_11931_MOESM1_ESM.docx]

**Supplementary Table 1**. The construction of HLI

| WCRF/AICR recommendations | Items | Categories | Score |
| --- | --- | --- | --- |
| 1. the diet rich in whole grains, vegetables, fruits, legumes* | Total intake of fruit and vegetable | <3 servings/day | 0 |
|  |  | ≥3~5 servings /day | 0.25 |
|  |  | ≥5 servings /day | 0.5 |
|  |  | （1 serving：1 piece of fresh fruit; 5 pieces of dried fruits; Boiled/Lettuce 3 tablespoons） |  |
|  | Whole grains intake | ≤ 2 servings /day | 0 |
|  |  | >2~<5.5 servings /day | 0.25 |
|  |  | ≥5.5 servings /day | 0.5 |
|  |  | （1 serving：bran/oatmeal/cereal congee 1 bowl/day; whole wheat bread 1 slice/day） |  |
| 1. red/processed meats in moderation | Red meat intake | >4 times/week | 0 |
|  |  | 2~4 times/week | 0.25 |
|  |  | ≤1 time/week | 0.5 |
|  | Red meat intake | >4 times/week | 0 |
|  |  | 2~4 times/week | 0.25 |
|  |  | ≤1 time/week | 0.5 |
| 3）Drink in moderation | Frequency of drinking | never | 1 |
|  |  | <5 times/week | 0.5 |
|  |  | ≥5 times/week | 0 |
| 4）More exercise | Physical activity | <600 MET-minute/week | 0 |
|  |  | ≥600~<3000 MET- minute/week | 0.5 |
|  |  | ≥3000 MET- minute/week | 1 |
| 5）Healthy weight | BMI ** | 18.5-24.9 kg/m2 | 0.5 |
|  |  | 25.0-29.9 kg/m2 | 0.25 |
|  |  | >29.9 kg/m2 | 0 |
|  | Waist circumference** | <80 cm | 0.5 |
|  |  | >80~<88 cm | 0.25 |
|  |  | ≥88 cm | 0 |
| 6）Limit consumption of fast foods and other processed foods high in fat, starches, or sugars | These five recommendations were not included in the construction of HLI * | | |
| 7）Limit consumption of sugary drinks |  |  |  |
| 8）No supplements used for cancer prevention |  |  |  |
| 9）Breastfeeding (for mothers) |  |  |  |
| 10）After cancer diagnosis, follow the recommended treatment guidelines |  |  |  |
| Smoking isn’t included in WCRF/AICR | smoking | Never | 1 |
|  |  | Former | 0.5 |
|  |  | Current | 0 |

* UK Biobank food frequency table does not include information on legume intake and the five recommended items mentioned above.

** BMI and waist circumference scores are reversed among premenopausal women (women under 55 years old or self-reported as premenopausal are classified as premenopausal women, while women over 55 years old or self-reported as postmenopausal are classified as postmenopausal women).

The composition and scoring of each component of HLI refer to Arthur et al. Adherence to Healthy Lifestyle Behavior, and Risk of Invasive Breast Cancer Among Women in the UK Biobank. *J Natl Cancer Inst*. 2020;112(9):893-901.

**Supplementary Table 2.** The levels of inflammation markers and the risk of breast cancer among women from the UK Biobank.

| Inflammation markers | No. | Incident cases | Multivariable-adjusted *HR* (95% *CI*) | |
| --- | --- | --- | --- | --- |
|  |  |  | Model 1^a^ | Model 2^b^ |
| CRP (mg/L) |  |  |  |  |
| Q1 | 62765 | 1845 | 1.00 (REF) | 1.00 (REF) |
| Q2 | 62006 | 2102 | **1.12 (1.05-1.19)** | **1.10 (1.03-1.17)** |
| Q3 | 61567 | 2247 | **1.19 (1.12-1.27)** | **1.15 (1.08-1.23)** |
| Q4 | 61978 | 2353 | **1.25 (1.18-1.33)** | **1.20 (1.12-1.28)** |
| *P*_trend_ |  |  | **<0.001** | **<0.001** |
| Standardized continuous |  |  | **1.03 (1.01-1.05)** | 1.01 (0.99-1.03) |
| NLR |  |  |  |  |
| Q1 | 63162 | 2060 | 1.00 (REF) | 1.00 (REF) |
| Q2 | 63191 | 2212 | **1.08 (1.02-1.15)** | 1.08 (1.02-1.15) |
| Q3 | 62852 | 2185 | **1.09 (1.03-1.16)** | 1.08 (1.02-1.15) |
| Q4 | 63056 | 2173 | **1.11 (1.04-1.18)** | 1.10 (1.03-1.17) |
| *P*_trend_ |  |  | **0.003** | 0.007 |
| Standardized continuous |  |  | **1.02 (1.01-1.04)** | 1.02 (1.00-1.04) |
| PLR |  |  |  |  |
| Q1 | 63072 | 2200 | 1.00 (REF) | 1.00 (REF) |
| Q2 | 63071 | 2122 | 0.96 (0.91-1.02) | 0.97 (0.91-1.03) |
| Q3 | 63052 | 2184 | 0.99 (0.94-1.05) | 1.00 (0.95-1.07) |
| Q4 | 63063 | 2124 | 0.97 (0.91-1.03) | 0.99 (0.93-1.05) |
| *P*_trend_ |  |  | 0.518 | 0.907 |
| Standardized continuous |  |  | 0.99 (0.97-1.02) | 1.00 (0.98-1.02) |
| LMR |  |  |  |  |
| Q1 | 63974 | 2281 | 1.00 (REF) | 1.00 (REF) |
| Q2 | 62116 | 2181 | 0.98 (0.92-1.04) | 0.98 (0.92-1.04) |
| Q3 | 63053 | 2155 | 0.95 (0.90-1.01) | 0.95 (0.89-1.01) |
| Q4 | 63018 | 2008 | **0.89 (0.84-0.95)** | **0.89 (0.84-0.94)** |
| *P*_trend_ |  |  | **<0.001** | **<0.001** |
| Standardized continuous |  |  | 0.99 (0.96-1.01) | 0.99 (0.96-1.01) |
| SII |  |  |  |  |
| Q1 | 63065 | 2049 | 1.00 (REF) | 1.00 (REF) |
| Q2 | 63070 | 2170 | 1.06 (1.00-1.13) | 1.05 (0.99-1.12) |
| Q3 | 63059 | 2186 | **1.09 (1.02-1.15)** | **1.07 (1.01-1.14)** |
| Q4 | 63064 | 2225 | **1.13 (1.06-1.20)** | **1.11 (1.04-1.18)** |
| *P*_trend_ |  |  | **<0.001** | **0.001** |
| Standardized continuous |  |  | **1.02 (1.00-1.04)** | **1.02 (1.00-1.04)** |
| CAR |  |  |  |  |
| Q1 | 56388 | 1664 | 1.00 (REF) | 1.00 (REF) |
| Q2 | 56389 | 1908 | **1.11 (1.03-1.18)** | **1.09 (1.02-1.16)** |
| Q3 | 56387 | 2056 | **1.18 (1.11-1.26)** | **1.14 (1.07-1.23)** |
| Q4 | 56388 | 2160 | **1.26 (1.18-1.34)** | **1.20 (1.12-1.29)** |
| *P*_trend_ |  |  | **<0.001** | **<0.001** |
| Standardized continuous |  |  | **1.03 (1.01-1.05)** | 1.01 (0.99-1.04) |
| CLR |  |  |  |  |
| Q1 | 60239 | 1789 | 1.00 (REF) | 1.00 (REF) |
| Q2 | 60245 | 2025 | **1.10 (1.03-1.17)** | **1.08 (1.01-1.15)** |
| Q3 | 60224 | 2222 | **1.20 (1.12-1.27)** | **1.16 (1.08-1.24)** |
| Q4 | 60229 | 2250 | **1.22 (1.15-1.30)** | **1.16 (1.09-1.25)** |
| *P*_trend_ |  |  | **<0.001** | **<0.001** |
| Standardized continuous |  |  | **1.02 (1.01-1.03)** | **1.02 (1.00-1.03)** |
| MHR |  |  |  |  |
| Q1 | 54842 | 1711 | 1.00 (REF) | 1.00 (REF) |
| Q2 | 54835 | 1900 | **1.12 (1.04-1.19)** | **1.10 (1.03-1.18)** |
| Q3 | 54840 | 1929 | **1.14 (1.07-1.21)** | **1.11 (1.04-1.19)** |
| Q4 | 54836 | 2015 | **1.20 (1.13-1.28)** | **1.15 (1.07-1.23)** |
| *P*_trend_ |  |  | **<0.001** | **<0.001** |
| Standardized continuous |  |  | 1.01 (1.00-1.01) | 1.01 (1.00-1.02) |
| NHR |  |  |  |  |
| Q1 | 54840 | 1711 | 1.00 (REF) | 1.00 (REF) |
| Q2 | 54837 | 1926 | **1.14 (1.07-1.21)** | **1.12 (1.05-1.20)** |
| Q3 | 54838 | 1926 | **1.16 (1.08-1.24)** | **1.12 (1.05-1.20)** |
| Q4 | 54838 | 1992 | **1.24 (1.16-1.32)** | **1.18 (1.10-1.26)** |
| *P*_trend_ |  |  | **<0.001** | **<0.001** |
| Standardized continuous |  |  | **1.05 (1.03-1.06)** | **1.04 (1.02-1.06)** |

a: model adjusted for the UK Biobank Assessment Center

b: model further adjusted for BMI, smoking, family history of breast cancer, number of births, oral contraceptive use, hormone replacement therapy, age at menarche, menopausal status at baseline.

Considering false-positive findings caused by multiple testing, biomarkers with *P* for trend<0.05/9 (the Bonferroni corrected threshold) were considered statistically significant.

**Supplementary Table 3.** The levels of inflammation markers and the risk of breast cancer by menopausal status

| Inflammation markers | No. | Incident cases | Premenopausal | |  | No. | Incident cases | Postmenopausal | |
| --- | --- | --- | --- | --- | --- | --- | --- | --- | --- |
|  |  |  | *HR* (95%*CI*) | |  |  |  | *HR* (95%*CI*) | |
|  |  |  | Model 1^a^ | Model 2^b^ |  |  |  | Model 1^a^ | Model 2^b^ |
| CRP (mg/L) |  |  |  |  |  |  |  |  |  |
| Q1 | 24359 | 450 | 1.00 (REF) | 1.00 (REF) |  | 38406 | 1395 | 1.00 (REF) | 1.00 (REF) |
| Q2 | 18275 | 347 | 1.04 (0.93-1.17) | 1.07 (0.96-1.21) |  | 43731 | 1755 | **1.16 (1.08-1.26)** | **1.13 (1.04-1.22)** |
| Q3 | 15968 | 276 | 1.08 (0.96-1.22) | 1.14 (1.01-1.29) |  | 45599 | 1971 | **1.26 (1.17-1.35)** | **1.18 (1.09-1.28)** |
| Q4 | 15854 | 270 | 1.00 (0.88-1.12) | 1.08 (0.94-1.24) |  | 46124 | 2083 | **1.37 (1.27-1.48)** | **1.26 (1.16-1.37)** |
| *P*_trend_ |  |  | 0.797 | 0.483 |  |  |  | **<0.001** | **<0.001** |
| Standardized continuous |  |  | 0.96 (0.92-1.01) | 0.97 (0.92-1.03) |  |  |  | **1.04 (1.02-1.07)** | **1.02 (1.00-1.05)** |
| LMR |  |  |  |  |  |  |  |  |  |
| Q1 | 20759 | 410 | 1.00 (REF) | 1.00 (REF) |  | 43215 | 1871 | 1.00 (REF) | 1.00 (REF) |
| Q2 | 18726 | 348 | 0.91 (0.81-1.02) | 0.92 (0.82-1.03) |  | 43390 | 1833 | 1.01 (0.94-1.08) | 1.00 (0.94-1.07) |
| Q3 | 18295 | 325 | **0.85 (0.76-0.96)** | **0.86 (0.77-0.97)** |  | 44758 | 1830 | 0.99 (0.93-1.06) | 0.99 (0.92-1.06) |
| Q4 | 17778 | 289 | **0.82 (0.73-0.93)** | **0.84 (0.75-0.95)** |  | 45240 | 1719 | **0.92 (0.86-0.99)** | **0.91 (0.85-0.98)** |
| *P*_trend_ |  |  | **0.001** | **0.003** |  |  |  | **0.014** | **0.007** |
| Standardized continuous |  |  | **0.91 (0.85-0.97)** | **0.92 (0.86-0.98)** |  |  |  | 1.00 (0.98-1.03) | 1.00 (0.98-1.03) |
| SII |  |  |  |  |  |  |  |  |  |
| Q1 | 14323 | 233 | 1.00 (REF) | 1.00 (REF) |  | 48742 | 1816 | 1.00 (REF) | 1.00 (REF) |
| Q2 | 16936 | 306 | 1.06 (0.93-1.22) | 1.06 (0.93-1.22) |  | 46134 | 1864 | 1.06 (0.99-1.13) | 1.05 (0.98-1.12) |
| Q3 | 20176 | 398 | 1.12 (0.98-1.27) | 1.11 (0.98-1.27) |  | 42883 | 1788 | 1.07 (1.00-1.14) | 1.06 (0.99-1.13) |
| Q4 | 24159 | 436 | 1.10 (0.97-1.25) | 1.09 (0.97-1.24) |  | 38905 | 1789 | **1.13 (1.06-1.22)** | **1.12 (1.04-1.20)** |
| *P*_trend_ |  |  | 0.156 | 0.217 |  |  |  | **0.001** | **0.002** |
| Standardized continuous |  |  | 1.03 (0.99-1.07) | 1.02 (0.98-1.06) |  |  |  | 1.02 (1.00-1.04) | 1.02 (1.00-1.04) |
| CAR |  |  |  |  |  |  |  |  |  |
| Q1 | 21885 | 405 | 1.00 (REF) | 1.00 (REF) |  | 34503 | 1259 | 1.00 (REF) | 1.00 (REF) |
| Q2 | 16496 | 309 | 1.05 (0.93-1.18) | 1.08 (0.96-1.22) |  | 39893 | 1599 | **1.15 (1.06-1.24)** | **1.11 (1.02-1.20)** |
| Q3 | 14567 | 244 | 1.06 (0.94-1.20) | 1.12 (0.99-1.28) |  | 41820 | 1812 | **1.25 (1.15-1.35)** | **1.17 (1.08-1.27)** |
| Q4 | 14447 | 260 | 1.01 (0.89-1.15) | 1.10 (0.96-1.27) |  | 41941 | 1900 | **1.37 (1.26-1.47)** | **1.25 (1.15-1.36)** |
| *P*_trend_ |  |  | 0.980 | 0.319 |  |  |  | **<0.001** | **<0.001** |
| Standardized continuous |  |  | 0.96 (0.91-1.01) | 0.97 (0.92-1.03) |  |  |  | **1.04 (1.02-1.07)** | 1.02 (1.00-1.05) |
| CLR |  |  |  |  |  |  |  |  |  |
| Q1 | 22483 | 421 | 1.00 (REF) | 1.00 (REF) |  | 37756 | 1368 | 1.00 (REF) | 1.00 (REF) |
| Q2 | 17962 | 319 | 1.02 (0.91-1.15) | 1.05 (0.94-1.19) |  | 42283 | 1706 | **1.14 (1.06-1.23)** | **1.10 (1.02-1.19)** |
| Q3 | 16151 | 302 | 1.10 (0.98-1.24) | **1.16 (1.03-1.31)** |  | 44073 | 1920 | **1.25 (1.16-1.35)** | **1.18 (1.09-1.27)** |
| Q4 | 15678 | 258 | 0.99 (0.88-1.12) | 1.06 (0.92-1.21) |  | 44551 | 1992 | **1.32 (1.23-1.42)** | **1.21 (1.12-1.31)** |
| *P*_trend_ |  |  | 0.755 | 0.625 |  |  |  | **<0.001** | **<0.001** |
| Standardized continuous |  |  | 0.98 (0.93-1.04) | 0.99 (0.94-1.05) |  |  |  | **1.03 (1.02-1.05)** | **1.03 (1.01-1.05)** |
| MHR |  |  |  |  |  |  |  |  |  |
| Q1 | 15204 | 254 | 1.00 (REF) | 1.00 (REF) |  | 39638 | 1457 | 1.00 (REF) | 1.00 (REF) |
| Q2 | 15995 | 292 | 1.07 (0.94-1.23) | 1.08 (0.95-1.24) |  | 38840 | 1608 | **1.13 (1.05-1.22)** | **1.10 (1.02-1.19)** |
| Q3 | 16693 | 290 | 1.08 (0.94-1.23) | 1.09 (0.95-1.25) |  | 38147 | 1639 | **1.16 (1.07-1.24)** | **1.11 (1.03-1.20)** |
| Q4 | 17616 | 342 | **1.18 (1.04-1.35)** | **1.22 (1.06-1.39)** |  | 37220 | 1673 | **1.20 (1.11-1.29)** | **1.12 (1.03-1.21)** |
| *P*_trend_ |  |  | **0.011** | **0.004** |  |  |  | **<0.001** | **0.017** |
| Standardized continuous |  |  | 1.00 (0.99-1.02) | 1.00 (0.99-1.02) |  |  |  | **1.13 (1.07-1.19)** | **1.08 (1.02-1.15)** |
| NHR |  |  |  |  |  |  |  |  |  |
| Q1 | 12749 | 194 | 1.00 (REF) | 1.00 (REF) |  | 42091 | 1517 | 1.00 (REF) | 1.00 (REF) |
| Q2 | 14850 | 286 | **1.18 (1.02-1.36)** | **1.18 (1.03-1.36)** |  | 39987 | 1640 | **1.12 (1.04-1.21)** | **1.09 (1.01-1.18)** |
| Q3 | 17284 | 325 | 1.13 (0.99-1.30) | **1.15 (1.00-1.33)** |  | 37554 | 1601 | **1.16 (1.07-1.250** | **1.10 (1.02-1.19)** |
| Q4 | 20625 | 373 | 1.09 (0.95-1.25) | 1.12 (0.97-1.29) |  | 34213 | 1619 | **1.29 (1.19-1.39)** | **1.20 (1.11-1.30)** |
| *P*_trend_ |  |  | 0.644 | 0.404 |  |  |  | **<0.001** | **<0.001** |
| Standardized continuous |  |  | 1.02 (0.98-1.05) | 1.02 (0.99-1.06) |  |  |  | **1.08 (1.05-1.11)** | **1.05 (1.02-1.08)** |

a: model adjusted for the UK Biobank Assessment Center

b: model further adjusted for BMI, smoking, family history of breast cancer, number of births, oral contraceptive use, hormone replacement therapy, age at menarche

Breast cancer cases in which women were younger than 55 years of age or self-reported premenopausal status at baseline, followed up to the age of 55 years, otherwise postmenopausal breast cancer.

**Supplementary Table 4.** The levels of inflammation markers and the risk of breast cancer grouped by 2 years entering the cohort

| Inflammation markers | No. | Incident cases | ≤2 years after attendance | |  | No. | Incident cases | >2 years after attendance | |
| --- | --- | --- | --- | --- | --- | --- | --- | --- | --- |
|  |  |  | *HR* (95%*CI*) | |  |  |  | *HR* (95%*CI*) | |
|  |  |  | Model 1^a^ | Model 2^b^ |  |  |  | Model 1^a^ | Model 2^b^ |
| CRP (mg/L) |  |  |  |  |  |  |  |  |  |
| Q1 | 62765 | 296 | 1.00 (REF) | 1.00 (REF) |  | 62469 | 1549 | 1.00 (REF) | 1.00 (REF) |
| Q2 | 62006 | 348 | 1.11 (0.95-1.30) | 1.12 (0.95-1.31) |  | 61658 | 1754 | **1.12 (1.04-1.20)** | **1.10 (1.02-1.17)** |
| Q3 | 61567 | 349 | 1.09 (0.93-1.27) | 1.10 (0.93-1.30) |  | 61218 | 1898 | **1.21 (1.13-1.30)** | **1.17 (1.09-1.25)** |
| Q4 | 61978 | 435 | **1.36 (1.17-1.58)** | **1.38 (1.17-1.62)** |  | 61543 | 1918 | **1.24 (1.15-1.32)** | **1.17 (1.08-1.26)** |
| *P*_trend_ |  |  | **<0.001** | **<0.001** |  |  |  | **<0.001** | **0.002** |
| Standardized continuous |  |  | **1.05 (1.01-1.10)** | 1.05 (1.00-1.09) |  |  |  | **1.02 (1.00-1.05)** | 1.01 (0.98-1.03) |
| LMR |  |  |  |  |  |  |  |  |  |
| Q1 | 63974 | 403 | 1.00 (REF) | 1.00 (REF) |  | 63571 | 1878 | 1.00 (REF) | 1.00 (REF) |
| Q2 | 62116 | 362 | 0.93 (0.80-1.07) | 0.93 (0.80-1.07) |  | 61754 | 1819 | 0.99 (0.93-1.06) | 0.99 (0.92-1.05) |
| Q3 | 63053 | 357 | 0.91 (0.79-1.05) | 0.91 (0.79-1.05) |  | 62696 | 1798 | 0.96 (0.90-1.03) | 0.95 (0.89-1.02) |
| Q4 | 63018 | 317 | **0.81 (0.70-0.94)** | **0.82 (0.71-0.95)** |  | 62701 | 1691 | **0.91 (0.85-0.97)** | **0.90 (0.84-0.96)** |
| *P*_trend_ |  |  | **0.007** | **0.008** |  |  |  | **0.002** | **0.001** |
| Standardized continuous |  |  | 0.94 (0.87-1.02) | 0.94 (0.87-1.02) |  |  |  | 1.00 (0.97-1.02) | 0.99 (0.97-1.02) |
| SII |  |  |  |  |  |  |  |  |  |
| Q1 | 63065 | 336 | 1.00 (REF) | 1.00 (REF) |  | 62729 | 1713 | 1.00 (REF) | 1.00 (REF) |
| Q2 | 63070 | 358 | 1.08 (0.93-1.25) | 1.07 (0.92-1.24) |  | 62712 | 1812 | 1.06 (0.99-1.13) | 1.05 (0.98-1.12) |
| Q3 | 63059 | 335 | 1.03 (0.88-1.20) | 1.02 (0.88-1.19) |  | 62724 | 1851 | **1.09 (1.02-1.17)** | **1.08 (1.02-1.16)** |
| Q4 | 63064 | 410 | **1.29 (1.12-1.49)** | **1.28 (1.10-1.48)** |  | 62654 | 1815 | **1.10 (1.03-1.17)** | **1.08 (1.01-1.16)** |
| *P*_trend_ |  |  | **0.001** | **0.001** |  |  |  | **0.007** | **0.022** |
| Standardized continuous |  |  | **1.05 (1.01-1.08)** | **1.05 (1.01-1.08)** |  |  |  | 1.02 (0.99-1.04) | 1.01 (0.99-1.04) |
| CAR |  |  |  |  |  |  |  |  |  |
| Q1 | 56388 | 263 | 1.00 (REF) | 1.00 (REF) |  | 56125 | 1401 | 1.00 (REF) | 1.00 (REF) |
| Q2 | 56389 | 312 | 1.11 (0.94-1.30) | 1.10 (0.93-1.30) |  | 56077 | 1596 | **1.11 (1.03-1.19)** | **1.08 (1.01-1.17)** |
| Q3 | 56387 | 315 | 1.08 (0.92-1.28) | 1.08 (0.91-1.28) |  | 56072 | 1741 | **1.20 (1.12-1.29)** | **1.16 (1.07-1.25)** |
| Q4 | 56388 | 404 | **1.40 (1.20-1.64)** | **1.39 (1.17-1.66)** |  | 55984 | 1756 | **1.23 (1.15-1.32)** | **1.16 (1.07-1.26)** |
| *P*_trend_ |  |  | **<0.001** | **<0.001** |  |  |  | **<0.001** | **0.004** |
| Standardized continuous |  |  | **1.05 (1.01-1.10)** | 1.04 (1.00-1.10) |  |  |  | **1.02 (1.00-1.05)** | 1.01 (0.98-1.03) |
| CLR |  |  |  |  |  |  |  |  |  |
| Q1 | 60239 | 285 | 1.00 (REF) | 1.00 (REF) |  | 59954 | 1504 | 1.00 (REF) | 1.00 (REF) |
| Q2 | 60245 | 310 | 1.03 (0.87-1.20) | 1.03 (0.87-1.21) |  | 59935 | 1715 | **1.11 (1.04-1.19)** | **1.09 (1.01-1.17)** |
| Q3 | 60224 | 358 | 1.16 (0.99-1.35) | 1.17 (0.99-1.38) |  | 59866 | 1864 | **1.21 (1.13-1.29)** | **1.16 (1.08-1.24)** |
| Q4 | 60229 | 424 | **1.37 (1.18-1.59)** | **1.39 (1.18-1.64)** |  | 59805 | 1826 | **1.19 (1.11-1.28)** | **1.12 (1.04-1.21)** |
| *P*_trend_ |  |  | **<0.001** | **<0.001** |  |  |  | **<0.001** | **0.050** |
| Standardized continuous |  |  | **1.02 (1.00-1.04)** | **1.02 (1.00-1.04)** |  |  |  | **1.02 (1.00-1.04)** | 1.01 (0.99-1.03) |
| MHR |  |  |  |  |  |  |  |  |  |
| Q1 | 54842 | 281 | 1.00 (REF) | 1.00 (REF) |  | 54561 | 1430 | 1.00 (REF) | 1.00 (REF) |
| Q2 | 54835 | 324 | 1.15 (0.98-1.34) | 1.14 (0.97-1.33) |  | 54511 | 1576 | **1.11 (1.03-1.19)** | **1.10 (1.02-1.18)** |
| Q3 | 54840 | 318 | 1.12 (0.95-1.31) | 1.10 (0.93-1.29) |  | 54522 | 1611 | **1.14 (1.06-1.23)** | **1.11 (1.04-1.20)** |
| Q4 | 54836 | 323 | 1.13 (0.96-1.32) | 1.10 (0.93-1.29) |  | 54513 | 1692 | **1.21 (1.13-1.30)** | **1.16 (1.08-1.25)** |
| *P*_trend_ |  |  | 0.238 | 0.462 |  |  |  | **<0.001** | **<0.001** |
| Standardized continuous |  |  | 1.01 (0.99-1.03) | 1.01 (0.98-1.03) |  |  |  | 1.01 (1.00-1.02) | 1.01 (1.00-1.02) |
| NHR |  |  |  |  |  |  |  |  |  |
| Q1 | 54840 | 288 | 1.00 (REF) | 1.00 (REF) |  | 54552 | 1423 | 1.00 (REF) | 1.00 (REF) |
| Q2 | 54837 | 335 | 1.16 (0.99-1.36) | 1.15 (0.98-1.35) |  | 54502 | 1591 | **1.13 (1.05-1.22)** | **1.11 (1.04-1.20)** |
| Q3 | 54838 | 310 | 1.09 (0.93-1.28) | 1.07 (0.91-1.26) |  | 54528 | 1616 | **1.17 (1.09-1.26)** | **1.14 (1.06-1.22)** |
| Q4 | 54838 | 313 | 1.13 (0.97-1.33) | 1.10 (0.93-1.30) |  | 54525 | 1679 | **1.25 (1.17-1.35)** | **1.20 (1.11-1.29)** |
| *P*_trend_ |  |  | 0.270 | 0.515 |  |  |  | **<0.001** | **<0.001** |
| Standardized continuous |  |  | 1.03 (0.98-1.08) | 1.02 (0.96-1.08) |  |  |  | **1.05 (1.03-1.07)** | **1.04 (1.02-1.06)** |

a: model adjusted for the UK Biobank Assessment Center.

b: model further adjusted for BMI, smoking, family history of breast cancer, number of births, oral contraceptive use, hormone replacement therapy, age at menarche, menopausal status at baseline.

**Supplementary Table 5.** The associations between HLI and inflammation markers.

| Inflammation markers | *β* (95%*CI*) | *P* value |
| --- | --- | --- |
| CRP | **-0.150 (-0.155, -0.145)** | <0.001 |
| LMR | 0.000 (-0.001, 0.002) | 0.599 |
| SII | **-0.018 (-0.020, -0.016)** | <0.001 |
| CAR | **-0.139 (-0.143, -0.134)** | <0.001 |
| CLR | **-0.122 (-0.127, -0.117)** | <0.001 |
| MHR | **-0.043 (-0.046, -0.041)** | <0.001 |
| NHR | **-0.055 (-0.057, -0.053)** | <0.001 |

Linear models adjusted for age at recruitment, UK Biobank Assessment Center

*HLI* Healthy Lifestyle Index

**Supplementary Table 6.** The associations between individual components of HLI and inflammation markers

| HLI components | Blood inflammation markers | | | | | | |
| --- | --- | --- | --- | --- | --- | --- | --- |
|  | *β* (95%*CI*) ^a^ | | | | | | |
|  | CRP | LMR | SII | CAR | CLR | MHR | NHR |
| Diet score | **-0.422 (-0.441, -0.404)** | **0.024 (0.017, 0.031)** | **-0.096 (-0.104, -0.088)** | **-0.431 (-0.450, -0.411)** | **-0.387 (-0.406, -0.368)** | **-0.080 (-0.088, -0.072)** | **-0.128 (-0.136, -0.120)** |
| Alcohol intake frequency |  |  |  |  |  |  |  |
| Never | 1.00 (REF) | 1.00 (REF) | 1.00 (REF) | 1.00 (REF) | 1.00 (REF) | 1.00 (REF) | 1.00 (REF) |
| <5 times/ week | **-0.207 (-0.221, -0.192)** | **-0.015 (-0.021, -0.010)** | **-0.009 (-0.015, -0.002)** | **-0.221 (-0.237, -0.205)** | **-0.186 (-0.201, -0.171)** | **-0.088 (-0.095, -0.082)** | **-0.112 (-0.118, -0.106)** |
| ≥5 times/week | **-0.413 (-0.430, -0.396)** | **-0.050 (-0.056, -0.043)** | **-0.016 (-0.024, -0.009)** | **-0.440 (-0.458, -0.421)** | **-0.366 (-0.384, -0.348)** | **-0.206 (-0.214, -0.198)** | **-0.269 (-0.277, -0.262)** |
| Physical activity (MET-min/wk) | **-0.000 (-0.000, -0.000)** | **-0.000 (-0.000, -0.000)** | **-0.000 (-0.000, -0.000)** | **-0.000 (-0.000, -0.000)** | **-0.000 (-0.000, -0.000)** | **-0.000 (-0.000, -0.000)** | **-0.000 (-0.000, -0.000)** |
| BMI (kg/m^2^) | **0.105 (0.104, 0.106)** | **0.003 (0.002, 0.003)** | **0.004 (0.004, 0.005)** | **0.107 (0.107, 0.108)** | **0.095 (0.094, 0.096)** | **0.024 (0.024, 0.025)** | **0.028 (0.027, 0.028)** |
| Waist Circumference (cm) | **0.042 (0.042, 0.043)** | **0.001 (0.001, 0.001)** | **0.002 (0.002, 0.002)** | **0.043 (0.043, 0.044)** | **0.038 (0.038, 0.039)** | **0.011 (0.011, 0.011)** | **0.012 (0.012, 0.012)** |
| Smoking |  |  |  |  |  |  |  |
| Never | 1.00 (REF) | 1.00 (REF) | 1.00 (REF) | 1.00 (REF) | 1.00 (REF) | 1.00 (REF) | 1.00 (REF) |
| Former | **0.055 (0.046, 0.065)** | 0.002 (-0.001, 0.006) | **-0.005 (-0.009, -0.001)** | **0.054 (0.044, 0.064)** | **0.034 (0.024, 0.044)** | **0.007 (0.003, 0.011)** | -0.002 (-0.006, 0.002) |
| Current | **0.253 (0.237, 0.268)** | **0.095 (0.089, 0.100)** | **0.023 (0.016, 0.030)** | **0.260 (0.244, 0.277)** | **0.065 (0.049, 0.081)** | **0.153 (0.146, 0.160)** | **0.257 (0.250, 0.263)** |

a: model adjusted for the UK Biobank Assessment Centers

*HLI* Health Lifestyle Index

*BMI* Body Mass Index

**Supplementary Table 7.** Independent and joint effects of HLI and inflammation markers on breast cancer risk.

| Models | Inflammation markers | | | | | | |
| --- | --- | --- | --- | --- | --- | --- | --- |
|  | *HR* (95% *CI*) | | | | | | |
|  | CRP | SII | LMR | CAR | CLR | MHR | NHR |
| No.* | 191425 | 194791 | 194712 | 173793 | 185993 | 169101 | 169101 |
| Independent effect models** |  |  |  |  |  |  |  |
| HLI → breast cancer | **0.90 (0.88-0.93)** | **0.90 (0.88-0.92)** | **0.90 (0.88-0.92)** | **0.90 (0.88-0.93)** | **0.90 (0.88-0.93)** | **0.90 (0.88-0.93)** | **0.90 (0.88-0.93)** |
| Mediator → breast cancer | **1.06 (1.04-1.09)** | **1.09 (1.04-1.15)** | **0.89 (0.84-0.95)** | **1.06 (1.04-1.09)** | **1.06 (1.03-1.08)** | **1.16 (1.10-1.23)** | **1.16 (1.10-1.24)** |
| Joint effect models*** |  |  |  |  |  |  |  |
| HLI → breast cancer, adjusted for mediator | **0.91 (0.89-0.93)** | **0.90 (0.88-0.93)** | **0.90 (0.88-0.92)** | **0.91 (0.89-0.93)** | **0.91 (0.89-0.93)** | **0.91 (0.88-0.93)** | **0.91 (0.89-0.93)** |
| Mediator → breast cancer, adjusted for HLI | **1.05 (1.02-1.07)** | **1.08 (1.03-1.14)** | **0.89 (0.84-0.95)** | **1.05 (1.02-1.07)** | **1.05 (1.02-1.07)** | **1.13 (1.07-1.20)** | **1.13 (1.06-1.20)** |

* *No.*: the number of populations both on missing data regarding HLI and corresponding inflammation marker

**Independent effect models: included HLI or the inflammation markers.

***Joint effect models: included HLI and the inflammation markers, which are mutually adjusted.

*HLI* Health Lifestyle Index

*No.* Number

**Supplementary Table 8.** The association between HLI and the risk of breast cancer by menopausal status.

|  | Premenopausal | | |  | Postmenopausal | | |
| --- | --- | --- | --- | --- | --- | --- | --- |
|  | No. | Incident cases | HR (95%CI) ^a^ |  | No. | Incident cases | HR (95%CI) ^b^ |
| HLI | 65874 | 1885 | **0.91 (0.84-0.97)** |  | 138007 | 5083 | **0.85 (0.82-0.89)** |

a in premenopausal women, HLI was constructed without body fatness (BMI and waist circumference)

b in postmenopausal women, HLI was constructed with body fatness (BMI and waist circumference)

The model was adjusted for the UK Biobank assessment centers, family history of breast cancer, number of births, oral contraceptive use, hormone replacement therapy, age at menarche.

**Supplementary Table 9.** The associations between individual components of HLI and breast cancer risk among overall, premenopausal, and postmenopausal women in UK Biobank

| HLI components | Overall | | |  | Premenopausal | | |  | Postmenopausal | | |
| --- | --- | --- | --- | --- | --- | --- | --- | --- | --- | --- | --- |
|  | No. | Incident cases | *HR* (95%*CI*) ^a^ |  | No. | Incident cases | *HR* (95%*CI*) ^a^ |  | No. | Incident cases | *HR* (95%*CI*) ^a^ |
| Diet score |  |  |  |  |  |  |  |  |  |  |  |
| 0-0.5 | 9886 | 331 | 1.00 (REF) |  | 3471 | 64 | 1.00 (REF) |  | 6415 | 267 | 1.00 (REF) |
| 0.5-1 | 168448 | 5917 | 1.03 (0.92-1.15) |  | 54881 | 1013 | 1.06 (0.86-1.31) |  | 113567 | 4904 | 1.01 (0.89-1.16) |
| 1-2 | 86713 | 2853 | 0.93 (0.83-1.04) |  | 21191 | 368 | 1.00 (0.80-1.24) |  | 65522 | 2485 | 0.91 (0.79-1.04) |
| Alcohol intake frequency |  |  |  |  |  |  |  |  |  |  |  |
| Never | 25372 | 808 | 1.00 (REF) |  | 6428 | 116 | 1.00 (REF) |  | 18944 | 692 | 1.00 (REF) |
| ≥ 1 times/month | 197636 | 6677 | 1.05 (0.98-1.13) |  | 62618 | 1135 | 1.01 (0.87-1.19) |  | 135018 | 5542 | 1.06 (0.97-1.15) |
| ≥ 5 times/week | 42746 | 1645 | **1.15 (1.06-1.26)** |  | 10714 | 199 | 1.13 (0.94-1.36) |  | 32032 | 1446 | **1.15 (1.05-1.27)** |
| Physical activity (MET-min/wk) |  |  |  |  |  |  |  |  |  |  |  |
| <600 | 38324 | 1359 | 1.00 (REF) |  | 12769 | 249 | 1.00 (REF) |  | 25555 | 1110 | 1.00 (REF) |
| ≥600-<3000 | 106733 | 3727 | 0.97 (0.91-1.03) |  | 34436 | 658 | 0.97 (0.86-1.09) |  | 72297 | 3069 | 0.97 (0.90-1.04) |
| ≥3000 | 60423 | 1919 | **0.87 (0.81-0.93)** |  | 17619 | 290 | 0.89 (0.78-1.02) |  | 42804 | 1629 | **0.86 (0.80-0.94)** |
| BMI (kg/m^2^) |  |  |  |  |  |  |  |  |  |  |  |
| 18.5-24.9 | 103218 | 3260 | 1.00 (REF) |  | 35286 | 704 | 1.00 (REF) |  | 67932 | 2556 | 1.00 (REF) |
| 25.0-29.9 | 97211 | 3441 | **1.09 (1.04-1.15)** |  | 26018 | 439 | 0.96 (0.87-1.06) |  | 71193 | 3002 | **1.15 (1.09-1.22)** |
| ≥ 30.0 | 62588 | 2346 | **1.18 (1.12-1.25)** |  | 17654 | 287 | 0.95 (0.85-1.06) |  | 44934 | 2059 | **1.28 (1.20-1.36)** |
| Waist Circumference (cm) |  |  |  |  |  |  |  |  |  |  |  |
| <80 | 102336 | 3092 | 1.00 (REF) |  | 36829 | 692 | 1.00 (REF) |  | 65507 | 2400 | 1.00 (REF) |
| >80-<88 | 66573 | 2371 | **1.15 (1.09-1.21)** |  | 18398 | 359 | 1.10 (0.97-1.25) |  | 48175 | 2012 | **1.17 (1.10-1.25)** |
| ≥88 | 96505 | 3655 | **1.22 (1.17-1.29)** |  | 24469 | 395 | 0.94 (0.83-1.06) |  | 72036 | 3260 | **1.30 (1.23-1.37)** |
| Smoking |  |  |  |  |  |  |  |  |  |  |  |
| Never | 158225 | 5223 | 1.00 (REF) |  | 50811 | 912 | 1.00 (REF) |  | 107414 | 4311 | 1.00 (REF) |
| Former | 82986 | 3047 | **1.08 (1.04-1.13)** |  | 20170 | 370 | 1.06 (0.96-1.16) |  | 62816 | 2677 | **1.09 (1.04-1.15)** |
| Current | 23788 | 840 | **1.13 (1.05-1.22)** |  | 8655 | 167 | 1.07 (0.94-1.23) |  | 15133 | 673 | **1.16 (1.06-1.26)** |

a: model adjusted for the UK Biobank Assessment Centers

Breast cancer cases in which women were younger than 55 years of age or self-reported premenopausal status at baseline, followed up to the age of 55 years, otherwise postmenopausal breast cancer.

*HLI* Health Lifestyle Index

*BMI* Body Mass Index

**Supplementary Table 10.** The mediation analysis of the inflammation markers in the association between HLI and breast cancer risk

| Models | Mediators | | | | | | | | | | | | |
| --- | --- | --- | --- | --- | --- | --- | --- | --- | --- | --- | --- | --- | --- |
|  | CRP | *P* value | SII | *P* value | CAR | *P* value | CLR | *P* value | MHR | *P* value | NHR | *P* value |  |
| Cox regression model for breast cancer |  |  |  |  |  |  |  |  |  |  |  |  |  |
| HLI | **0.903 (0.880, 0.927)** | **<0.001** | **0.903 (0.881, 0.925)** | **<0.001** | **0.909 (0.886, 0.932)** | **<0.001** | **0.913 (0.890, 0.936)** | **<0.001** | **0.906 (0.883, 0.930)** | **<0.001** | **0.908 (0.885, 0.932)** | **<0.001** |  |
| Mediator | **1.048 (1.024, 1.072)** | **<0.001** | **1.039 (1.014, 1.065)** | **0.002** | **1.054 (1.027, 1.082)** | **<0.001** | **1.046 (1.023, 1.071)** | **<0.001** | **1.060 (1.032, 1.088)** | **<0.001** | **1.055 (1.027, 1.083)** | **<0.001** |  |
| HLI*mediator | 1.019 (0.996, 1.041) | 0.100 | 1.011 (0.987, 1.036) | 0.367 | 1.018 (0.993, 1.044) | 0.158 | 1.020 (0.997, 1.043) | 0.084 | 1.014 (0.988, 1.040) | 0.289 | 1.010 (0.984, 1.036) | 0.461 |  |
| Linear model for the mediators |  |  |  |  |  |  |  |  |  |  |  |  |  |
| HLI | **-0.150 (-0.154, -0.145)** | **<0.001** | **-0.037 (-0.042, -0.033)** | **<0.001** | **-0.138 (-0.143, -0.134)** | **<0.001** | **-0.121 (-0.126, -0.116)** | **<0.001** | **-0.096 (-0.100, -0.091)** | **<0.001** | **-0.126 (-0.130, -0.121)** | **<0.001** |  |
| 4-way decomposition of mediation |  |  |  |  |  |  |  |  |  |  |  |  |  |
| Controlled direct effect | **-0.096 (-0.120, -0.073)** | **<0.001** | **-0.097 (-0.118, -0.075)** | **<0.001** | **-0.092 (-0.116, -0.069)** | **<0.001** | **-0.089 (-0.113, -0.066)** | **<0.001** | **-0.094 (-0.117, -0.070)** | **<0.001** | **-0.091 (-0.115, -0.068)** | **<0.001** |  |
| Reference interaction | 0.002 (-0.002, 0.005) | 0.214 | 0.001 (-0.002, 0.005) | 0.549 | -0.002 (-0.005, 0.002) | 0.340 | -0.006 (-0.016, 0.003) | 0.184 | 0.001 (-0.001, 0.002) | 0.419 | 0.001 (-0.003, 0.004) | 0.690 |  |
| Mediated interaction | -0.0018 (-0.005, 0.001) | 0.215 | -0.000 (-0.001, 0.001) | 0.570 | -0.002 (-0.005, 0.001) | 0.316 | -0.002 (-0.004, 0.001) | 0.184 | -0.001 (-0.003, 0.002) | 0.545 | -0.000 (-0.003, 0.002) | 0.744 |  |
| Pure indirect effect | **-0.007 (-0.010, -0.0035)** | **<0.001** | **-0.001 (-0.002, -0.000)** | **0.003** | **-0.007 (-0.011, -0.004)** | **<0.001** | **-0.005 (-0.008, -0.003)** | **<0.001** | **-0.006 (-0.008, -0.003)** | **<0.001** | **-0.007 (-0.010, -0.0034)** | **<0.001** |  |
| Overall proportion of mediation | 8.50% | **<0.001** | 1.71% | **0.012** | 8.66% | **<0.001** | 6.91% | **<0.001** | 6.27% | **0.001** | 7.33% | **0.003** |  |
| Overall proportion of interaction | 0.16% | 0.533 | 0.86% | 0.548 | 3.04% | 0.313 | 7.80% | 0.174 | 0.17% | 0.761 | 0.23% | 0.481 |  |

Models adjusted for age at recruitment, number of births, family history of breast cancer, oral contraceptive use, hormone replacement therapy, and age at menarche.

*HLI* Health Lifestyle Index

**Supplementary Table 11.** Mediating effects of inflammation markers on the association between diet score and breast cancer risk

|  | CRP | *P* value | SII | *P* value | CAR | *P* value | CLR | *P* value | MHR | *P* value | NHR | *P* value |
| --- | --- | --- | --- | --- | --- | --- | --- | --- | --- | --- | --- | --- |
| Cox regression model for breast cancer |  |  |  |  |  |  |  |  |  |  |  |  |
| Diet score | **0.960 (0.938, 0.983)** | **0.001** | **0.959 (0.939, 0.980)** | **<0.001** | **0.969 (0.947, 0.991)** | **0.006** | **0.971 (0.950, 0.993)** | **0.009** | **0.965 (0.943, 0.987)** | **0.002** | **0.967 (0.945, 0.989)** | **0.003** |
| Mediator | **1.064 (1.043, 1.085)** | **<0.001** | **1.037 (1.015, 1.059)** | **0.001** | **1.071 (1.047, 1.095)** | **<0.001** | **1.058 (1.037, 1.079)** | **<0.001** | **1.066 (1.042, 1.091)** | **<0.001** | **1.066 (1.042, 1.091)** | **<0.001** |
| Diet score*mediator | 1.017 (0.997, 1.037) | 0.090 | **1.028 (1.006, 1.050)** | **0.010** | 1.012 (0.990, 1.034) | 0.303 | **1.022 (1.002, 1.042)** | **0.032** | 1.014 (0.992, 1.038) | 0.213 | 1.018 (0.995, 1.041) | 0.129 |
| Linear model for the mediators |  |  |  |  |  |  |  |  |  |  |  |  |
| Diet score | **-0.096 (-0.100, -0.092)** | **<0.001** | **-0.046 (-0.050, -0.042)** | **<0.001** | **-0.089 (-0.093, -0.084)** | **<0.001** | **-0.088 (-0.093, -0.084)** | **<0.001** | **-0.040 (-0.044, -0.036)** | **<0.001** | **-0.067 (-0.071, -0.062)** | **<0.001** |
| 4-way decomposition of mediation |  |  |  |  |  |  |  |  |  |  |  |  |
| Controlled direct effect | **-0.039 (-0.061, -0.017)** | **<0.001** | **-0.041 (-0.061, -0.020)** | **<0.001** | **-0.032 (-0.054, -0.010)** | **0.005** | **-0.030 (-0.051, -0.008)** | **0.008** | **-0.035 (-0.057, -0.013)** | **0.002** | **-0.033 (-0.055, -0.011)** | **0.003** |
| Reference interaction | 0.003 (-0.001, 0.007) | 0.145 | **0.004 (0.0005, 0.008)** | **0.026** | -0.001 (-0.004, 0.002) | 0.416 | **-0.008 (-0.016, -0.00007)** | **0.048** | 0.001 (-0.001, 0.002) | 0.313 | 0.003 (-0.001, 0.006) | 0.194 |
| Mediated interaction | -0.001 (-0.003, 0.0004) | 0.138 | **-0.001 (-0.002, -0.0002)** | **0.017** | -0.001 (-0.003, 0.001) | 0.402 | -0.002 (-0.003, -0.00001) | 0.051 | -0.0005 (-0.001, 0.0004) | 0.296 | -0.001 (-0.002, 0.0005) | 0.184 |
| Pure indirect effect | **-0.006 (-0.008, -0.004)** | **<0.001** | **-0.002 (-0.003, -0.0007)** | **0.001** | **-0.006 (-0.008, -0.004)** | **<0.001** | **-0.005 (-0.007, -0.003)** | **<0.001** | **-0.0025 (-0.0035, -0.002)** | **<0.001** | **-0.004 (-0.006, -0.003)** | **<0.001** |
| Overall proportion of mediation | 16.58% | **<0.001** | 7.17% | **0.009** | 17.20% | **0.002** | 14.99% | **0.001** | 7.98% | **0.007** | 14.66% | **0.010** |
| Overall proportion of interaction | 3.81% | 0.142 | 7.39% | 0.081 | 4.98% | 0.399 | 21.67% | 0.056 | 0.45% | 0.473 | 4.23% | 0.250 |

Models adjusted for age at recruitment, number of births, family history of breast cancer, oral contraceptive use, hormone replacement therapy, and age at menarche.

**Supplementary Table 12.** Mediating effects of inflammation markers on the association between physical activity and breast cancer risk

| Models | Mediators | | | | | | | | | | | |
| --- | --- | --- | --- | --- | --- | --- | --- | --- | --- | --- | --- | --- |
|  | CRP | *P* value | SII | *P* value | CAR | *P* value | CLR | *P* value | MHR | *P* value | NHR | *P* value |
| Cox regression model for breast cancer |  |  |  |  |  |  |  |  |  |  |  |  |
| Physical activity | **0.957 (0.932, 0.983)** | **0.001** | **0.950 (0.926, 0.974)** | **<0.001** | **0.955 (0.930, 0.980)** | **0.001** | **0.959 (0.933, 0.984)** | **0.002** | **0.953 (0.927, 0.979)** | **<0.001** | **0.954 (0.929, 0.981)** | **0.001** |
| Mediator | **1.059 (1.035, 1.083)** | **<0.001** | **1.039 (1.014, 1.065)** | **0.002** | **1.066 (1.039, 1.094)** | **<0.001** | **1.054 (1.031, 1.079)** | **<0.001** | **1.067 (1.039, 1.095)** | **<0.001** | **1.064 (1.037, 1.093)** | **<0.001** |
| Physical activity*mediator | 1.007 (0.984, 1.031) | 0.545 | 0.984 (0.960, 1.009) | 0.221 | 1.008 (0.982, 1.036) | 0.539 | 0.999 (0.976, 1.023) | 0.944 | 1.020 (0.993, 1.048) | 0.147 | 0.998 (0.972, 1.026) | 0.906 |
| Linear model for the mediators |  |  |  |  |  |  |  |  |  |  |  |  |
| Physical activity | **-0.094 (-0.099, -0.089)** | **<0.001** | **-0.029 (-0.033, -0.024)** | **<0.001** | **-0.087 (-0.092, -0.082)** | **<0.001** | **-0.089 (-0.094, -0.084)** | **<0.001** | **-0.062 (-0.067, -0.057)** | **<0.001** | **-0.078 (-0.083, -0.073)** | **<0.001** |
| 4-way decomposition of mediation |  |  |  |  |  |  |  |  |  |  |  |  |
| Controlled direct effect | **-0.043 (-0.068, -0.017)** | **0.001** | **-0.050 (-0.074, -0.026)** | **<0.001** | -0.046 (-0.071, -0.020) | **<0.001** | **-0.043 (-0.069, -0.016)** | **0.001** | **-0.047 (-0.073, -0.022)** | **<0.001** | **-0.045 (-0.071, -0.020)** | **0.001** |
| Reference interaction | 0.0009 (-0.003, 0.005) | 0.664 | -0.002 (-0.006, 0.001) | 0.150 | -0.0006 (-0.004, 0.003) | 0.733 | 0.001 (-0.009, 0.012) | 0.787 | 0.001 (0.0008, 0.003) | 0.254 | -0.0006 (-0.005, 0.004) | 0.768 |
| Mediated interaction | -0.0004 (-0.003, 0.002) | 0.688 | 0.0005 (-0.0002, 0.001) | 0.169 | -0.0004 (-0.003, 0.002) | 0.696 | 0.0003 (-0.002,0.002) | 0.794 | -0.001 (-0.003, 0.001) | 0.223 | 0.0003 (-0.002, 0.002) | 0.740 |
| Pure indirect effect | **-0.005 (-0.007, -0.003)** | **<0.001** | **-0.001 (-0.002, -0.0004)** | **0.003** | **-0.006 (-0.008, -0.003)** | **<0.001** | **-0.005 (-0.007, -0.003)** | **<0.001** | **-0.004 (-0.006, -0.002)** | **<0.001** | **-0.005 (-0.007, -0.003)** | **<0.001** |
| Overall proportion of mediation | 12.13% | **0.004** | 1.17% | 0.250 | 11.48% | **0.004** | 9.77% | **0.014** | 9.80% | **0.005** | 8.98% | **0.023** |
| Overall proportion of interaction | 0.94% | 0.633 | 3.68% | 0.150 | 1.89% | 0.714 | 3.79% | 0.791 | 0.31% | 0.615 | 0.57% | 0.793 |

Models adjusted for age at recruitment, number of births, family history of breast cancer, oral contraceptive use, hormone replacement therapy, and age at menarche.

**Supplementary Table 13.** Mediating effects of inflammation markers on the association between BMI and breast cancer risk

| Models | Mediators | | | | | | | | | | | |
| --- | --- | --- | --- | --- | --- | --- | --- | --- | --- | --- | --- | --- |
|  | CRP | *P* value | SII | *P* value | CAR | *P* value | CLR | *P* value | MHR | *P* value | NHR | *P* value |
| Cox regression model for breast cancer |  |  |  |  |  |  |  |  |  |  |  |  |
| BMI | **1.072 (1.040, 1.105)** | **<0.001** | **1.071 (1.049, 1.094)** | **<0.001** | **1.064 (1.035, 1.095)** | **<0.001** | **1.062 (1.037, 1.088)** | **<0.001** | **1.065 (1.040, 1.090)** | **<0.001** | **1.066 (1.040, 1.093)** | **<0.001** |
| Mediator | **1.043 (1.020, 1.067)** | **<0.001** | **1.036 (1.014, 1.059)** | **0.001** | **1.048 (1.021, 1.076)** | **<0.001** | **1.037 (1.014, 1.060)** | **0.001** | **1.052 (1.027, 1.077)** | **<0.001** | **1.051 (1.026, 1.077)** | **<0.001** |
| BMI*mediator | **0.976 (0.958, 0.994)** | **0.010** | 0.989 (0.970, 1.009) | 0.297 | **0.970 (0.950, 0.991)** | **0.005** | **0.978 (0.960, 0.997)** | **0.022** | 0.985 (0.964, 1.006) | 0.165 | **0.979 (0.958, 1.000)** | **0.049** |
| Linear model for the mediators |  |  |  |  |  |  |  |  |  |  |  |  |
| BMI | **0.543 (0.539, 0.547)** | **<0.001** | **0.049 (0.045, 0.053)** | **<0.001** | **0.502 (0.498, 0.505)** | **<0.001** | **0.490 (0.486, 0.494)** | **<0.001** | **0.274 (0.270, 0.278)** | **<0.001** | **0.324 (0.320, 0.328)** | **<0.001** |
| 4-way decomposition of mediation |  |  |  |  |  |  |  |  |  |  |  |  |
| Controlled direct effect | **0.071 (0.039, 0.104)** | **<0.001** | **0.071 (0.049, 0.093)** | **<0.001** | **0.065 (0.035, 0.095)** | **<0.001** | **0.063 (0.037, 0.089)** | **<0.001** | **0.065 (0.040, 0.090)** | **<0.001** | **0.066 (0.039, 0.092)** | **<0.001** |
| Reference interaction | **-0.005 (-0.009, -0.001)** | **0.014** | -0.001 (-0.005, 0.002) | 0.387 | **0.004 (0.000, 0.007)** | **0.027** | **0.009 (0.000, 0.018)** | **0.047** | -0.001 (-0.002, 0.0003) | 0.183 | -0.003 (-0.007, 0.0002) | 0.068 |
| Mediated interaction | **-0.013 (-0.024, -0.002)** | **0.020** | -0.000 (-0.001, 0.001) | 0.413 | **-0.015 (-0.026, -0.003)** | **0.011** | **-0.010 (-0.020, -0.001)** | **0.039** | -0.004 (-0.010, 0.003) | 0.259 | -0.007 (-0.014, 0.001) | 0.086 |
| Pure indirect effect | **0.023 (0.010, 0.036)** | **<0.001** | **0.002 (0.001, 0.003)** | **0.001** | **0.024 (0.011, 0.037)** | **<0.001** | **0.018 (0.007, 0.029)** | **0.002** | **0.014 (0.007, 0.021)** | **<0.001** | **0.016 (0.008, 0.024)** | **<0.001** |
| Overall proportion of mediation | 13.36% | 0.266 | 1.82% | **0.094** | 11.77% | 0.347 | 9.30% | 0.364 | 13.84% | **0.033** | 13.45% | 0.090 |
| Overall proportion of interaction | 23.36% | **0.020** | 2.57% | 0.403 | 14.25% | **0.010** | 1.97% | **0.020** | 5.76% | 0.258 | 13.66% | 0.105 |

Models adjusted for age at recruitment, number of births, family history of breast cancer, oral contraceptive use, hormone replacement therapy, and age at menarche.

*BMI* Body Mass Index

**Supplementary Table 14.** Mediating effects of inflammation markers on the association between WC and breast cancer risk

| Models | Mediators | | | | | | | | | | | |
| --- | --- | --- | --- | --- | --- | --- | --- | --- | --- | --- | --- | --- |
|  | CRP | *P* value | SII | *P* value | CAR | *P* value | CLR | *P* value | MHR | *P* value | NHR | *P* value |
| Cox regression model for breast cancer |  |  |  |  |  |  |  |  |  |  |  |  |
| Waist circumference | **1.094 (1.064, 1.125)** | **<0.001** | **1.086 (1.064, 1.109)** | **<0.001** | **1.082 (1.053, 1.111)** | **<0.001** | **1.079 (1.054, 1.105)** | **<0.001** | **1.078 (1.053, 1.104)** | **<0.001** | **1.081 (1.054, 1.107)** | **<0.001** |
| Mediator | **1.035 (1.012, 1.059)** | **0.002** | **1.035 (1.013, 1.058)** | **0.002** | **1.041 (1.015, 1.068)** | **0.002** | **1.031 (1.008, 1.054)** | **0.007** | **1.046 (1.021, 1.071)** | **<0.001** | **1.045 (1.020, 1.071)** | **<0.001** |
| Waist circumference*mediator | **0.972 (0.954, 0.990)** | **0.003** | 0.985 (0.965, 1.006) | 0.153 | **0.966 (0.945, 0.987)** | **0.001** | **0.975 (0.957, 0.994)** | **0.010** | 0.988 (0.967, 1.010) | 0.285 | **0.977 (0.956, 0.998)** | **0.032** |
| Linear model for the mediators |  |  |  |  |  |  |  |  |  |  |  |  |
| Waist circumference | **0.531 (0.527, 0.535)** | **<0.001** | **0.058 (0.054, 0.061)** | **<0.001** | **0.490 (0.486, 0.493)** | **<0.001** | **0.477 (0.473, 0.481)** | **<0.001** | **0.296 (0.292, 0.300)** | **<0.001** | **0.351 (0.347, 0.355)** | **<0.001** |
| 4-way decomposition of mediation |  |  |  |  |  |  |  |  |  |  |  |  |
| Controlled direct effect | **0.093 (0.062, 0.124)** | **<0.001** | **0.086 (0.063, 0.108)** | **<0.001** | **0.082 (0.053, 0.111)** | **<0.001** | **0.080 (0.055, 0.106)** | **<0.001** | **0.078 (0.053, 0.104)** | **<0.001** | **0.080 (0.054, 0.106)** | **<0.001** |
| Reference interaction | **-0.007 (-0.112, -0.002)** | **0.005** | -0.002 (-0.005, 0.001) | 0.215 | **0.003 (0.001, 0.006)** | **0.016** | **0.010 (0.001, 0.018)** | **0.025** | -0.001 (-0.002, 0.001) | 0.369 | **-0.004 (-0.008, -0.000)** | **0.048** |
| Mediated interaction | **-0.015 (-0.026, -0.004)** | **0.007** | -0.001 (-0.002, 0.001) | 0.240 | **-0.017 (-0.028, -0.006)** | **0.004** | **-0.012 (-0.022, -0.002)** | **0.019** | -0.003 (-0.010, 0.004) | 0.431 | -0.008 (-0.016, 0.000) | 0.059 |
| Pure indirect effect | **0.019 (0.006, 0.031)** | **0.003** | **0.002 (0.001, 0.003)** | **0.002** | **0.020 (0.007, 0.033)** | **0.002** | **0.015 (0.004, 0.025)** | **0.007** | **0.013 (0.006, 0.021)** | **<0.001** | **0.016 (0.007, 0.024)** | **<0.001** |
| Overall proportion of mediation | 4.01% | 0.677 | 1.45% | 0.170 | 3.35% | 0.746 | 3.02% | 0.715 | 12.01% | **0.036** | 9.26% | 0.199 |
| Overall proportion of interaction | 24.48% | **0.009** | 3.30% | 0.237 | 7.14% | **0.005** | 2.38% | **0.012** | 3.91% | 0.429 | 14.38% | 0.082 |

Models adjusted for age at recruitment, number of births, family history of breast cancer, oral contraceptive use, hormone replacement therapy, and age at menarche.

**Supplementary Table 15.** Mediating effects of inflammation markers on the association between smoking and breast cancer risk

| Models | Mediators | | | | | | | | | | | |
| --- | --- | --- | --- | --- | --- | --- | --- | --- | --- | --- | --- | --- |
|  | CRP | *P* value | SII | *P* value | CAR | *P* value | CLR | *P* value | MHR | *P* value | NHR | *P* value |
| Cox regression model for breast cancer |  |  |  |  |  |  |  |  |  |  |  |  |
| Smoking | **1.081 (1.032, 1.133)** | **0.001** | **1.083 (1.038, 1.131)** | **<0.001** | **1.080 (1.032, 1.130)** | **0.001** | **1.087 (1.039, 1.136)** | **<0.001** | **1.074 (1.026, 1.124)** | **0.002** | **1.071 (1.023, 1.121)** | **0.003** |
| Mediator | **1.059 (1.032, 1.087)** | **<0.001** | **1.057 (1.028, 1.087)** | **<0.001** | **1.064 (1.033, 1.097)** | **<0.001** | **1.057 (1.029, 1.085)** | **<0.001** | **1.063 (1.031, 1.096)** | **<0.001** | **1.071 (1.038, 1.105)** | **<0.001** |
| Smoking*mediator | 1.013 (0.974, 1.053) | 0.525 | 0.959 (0.919, 1.001) | 0.058 | 1.017 (0.972, 1.064) | 0.469 | 1.007 (0.968, 1.048) | 0.730 | 1.005 (0.960, 1.053) | 0.821 | 0.995 (0.950, 1.042) | 0.822 |
| Linear model for the mediators |  |  |  |  |  |  |  |  |  |  |  |  |
| Smoking | **0.098 (0.090, 0.107)** | **<0.001** | **0.003 (-0.005, 0.011)** | **0.482** | **0.090 (0.081, 0.098)** | **<0.001** | **0.040 (0.031, 0.049)** | **<0.001** | **0.087 (0.078, 0.095)** | **<0.001** | **0.126 (0.118, 0.135)** | **<0.001** |
| 4-way decomposition of mediation |  |  |  |  |  |  |  |  |  |  |  |  |
| Controlled direct effect | **0.081 (0.031, 0.131)** | **0.002** | **0.083 (0.037, 0.129)** | **<0.001** | **0.081 (0.031, 0.131)** | **0.001** | **0.089 (0.039, 0.138)** | **<0.001** | **0.074 (0.025, 0.123)** | **0.003** | **0.071 (0.022, 0.120)** | **0.005** |
| Reference interaction | 0.003 (-0.004, 0.010) | 0.402 | -0.006 (-0.013, 0.001) | 0.070 | -0.004 (-0.012, 0.004) | 0.307 | -0.006 (-0.024, 0.013) | 0.551 | -0.00002 (-0.001, 0.001) | 0.940 | -0.0002 (-0.006, 0.005) | 0.930 |
| Mediated interaction | 0.002 (-0.002, 0.006) | 0.368 | -0.0001 (-0.0005, 0.0002) | 0.514 | 0.002 (-0.002, 0.006) | 0.336 | 0.0005 (-0.001, 0.002) | 0.563 | 0.001 (-0.003, 0.005) | 0.676 | -0.0001 (-0.006, 0.006) | 0.971 |
| Pure indirect effect | **0.006 (0.003, 0.008)** | **<0.001** | **0.000 (-0.000, 0.001)** | **0.489** | **0.006 (0.003, 0.008)** | **<0.001** | **0.002 (0.001, 0.003)** | **<0.001** | **0.005 (0.003, 0.008)** | **<0.001** | **0.009 (0.005, 0.013)** | **<0.001** |
| Overall proportion of mediation | 8.20% | **0.005** | 0.06% | 0.594 | 9.02% | **0.009** | 3.13% | **0.013** | 7.75% | **0.012** | 10.84% | **0.010** |
| Overall proportion of interaction | 5.39% | 0.415 | 8.11% | 0.136 | 2.29% | 0.315 | 5.90% | 0.563 | 1.08% | 0.724 | 0.46% | 0.952 |

Models adjusted for age at recruitment, number of births, family history of breast cancer, oral contraceptive use, hormone replacement therapy, and age at menarche.


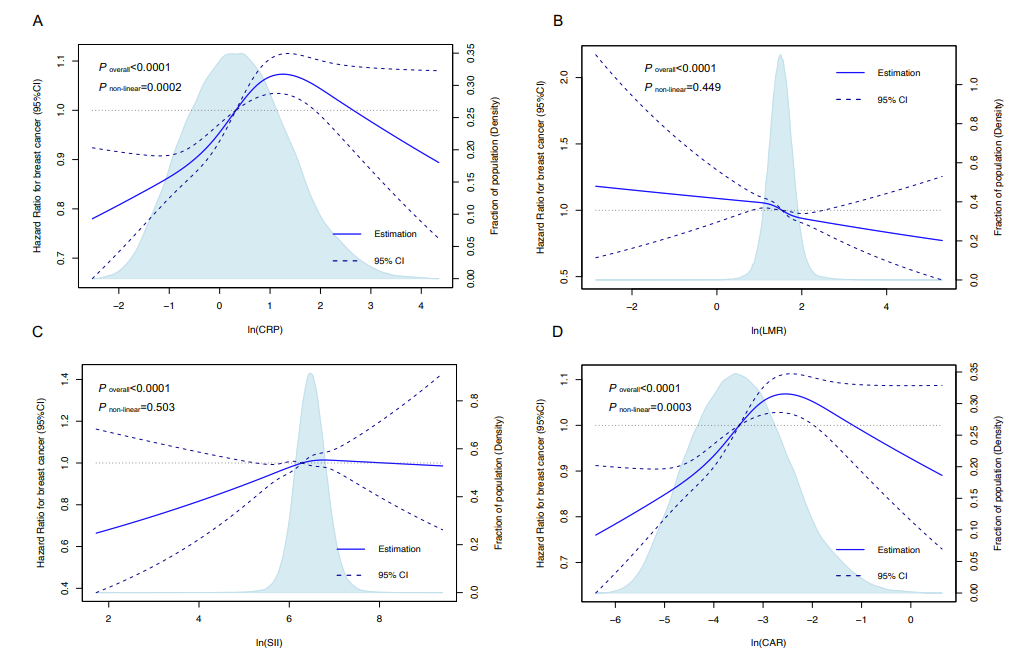

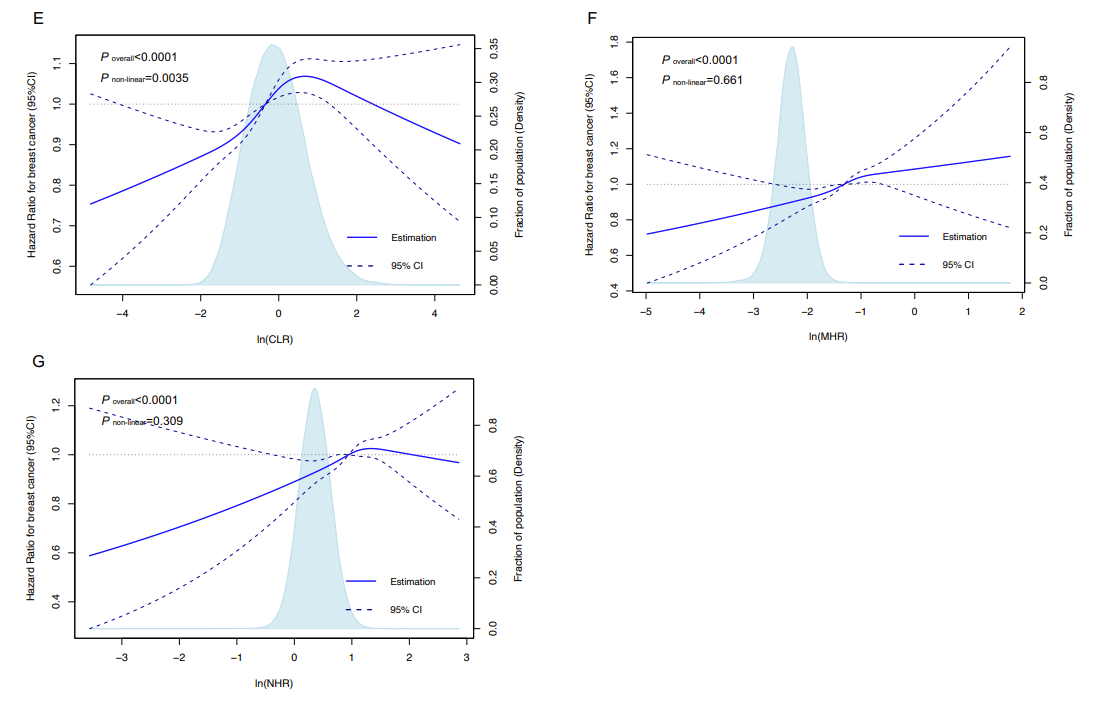


**Supplementary Figure 1.** The associations between levels of CRP, LMR, SII, CAR, CLR, MHR and NHR and breast cancer were evaluated on a continuous scale with restricted cubic spline curves based on cox regression with four knots. Solid lines are multivariable adjusted odds ratios, with dashed lines showing 95% confidence intervals. Blue curves show the fraction of breast cancer with different levels of inflammation markers.
